# Delayed application of N fertilizer mitigates the carbon emissions of pea/maize intercropping *via* altering soil microbial diversity

**DOI:** 10.3389/fmicb.2022.1002009

**Published:** 2022-09-23

**Authors:** Ke Xu, Falong Hu, Zhilong Fan, Wen Yin, Yining Niu, Qiming Wang, Qiang Chai

**Affiliations:** ^1^College of Agronomy, Gansu Agricultural University, Lanzhou, China; ^2^State Key Laboratory of Aridland Crop Science, Lanzhou, China

**Keywords:** N fertilizer postponing application, pea/maize intercropping, soil properties, soil microbial diversity, carbon emission

## Abstract

Strategies to reduce carbon emissions have been a hotspot in sustainable agriculture production. The delayed N fertilizer application had the potential to reduce carbon emissions in pea (*Pisum sativum* L.)/maize (*Zea mays* L.) intercropping, but its microbial mechanism remains unclear. In this study, we investigated the effects of delayed N fertilizer application on CO_2_ emissions and soil microbial diversity in pea/maize intercropping. The soil respiration (Rs) rates of intercropped pea and intercropped maize were decreased by 24.7% and 25.0% with delayed application of N fertilizer, respectively. The total carbon emissions (TCE) of the pea/maize intercropping system were also decreased by 21.1% compared with that of the traditional N fertilizer. Proteobacteria, Bacteroidota, and Chloroflexi were dominant bacteria in pea and maize strips. Heatmap analysis showed that the soil catalase activity at the pea flowering stage and the soil $Ν{Η}_{4}^{+}-Ν$ at the maize silking stage contributed more to the variations of bacterial relative abundances than other soil properties. Network analysis demonstrated that Rs was positively related to the relative abundance of Proteobacteria and Bacteroidota, while negatively related to the relative abundance of Chloroflexi in the pea/maize intercropping system. Overall, our results suggested that the delayed application of N fertilizer combined with the pea/maize intercropping system altered soil bacterial community diversity, thereby providing novel insights into connections between soil microorganisms and agricultural carbon emissions.

## Introduction

Agricultural production activities are the major source of CO_2_, N_2_O, and CH_4_ emissions, accounting for approximately 14% of the total worldwide (IPCC, 2014). Although agricultural N_2_O and CH_4_ emissions contribute a higher percentage than CO_2_, reducing CO_2_ to net-zero by 2050 is imperative due to its long-lasting effect (IPCC, 2018; Lynch et al., 2021). Additionally, CO_2_ is the main source of agricultural GHGs in arid regions of China (Qu et al., 2013). Therefore, exploring ameliorated agronomic practices with low carbon emissions is an urgent technique for the sustainability of modern agriculture.

Agricultural CO_2_ emissions can be decreased by optimizing the management practices of cropping systems. Intercropping, cultivating multiple crop species in a field, can boost crop productivity (Li et al., 2001), improve resource use efficiency (Yin et al., 2015; Gou et al., 2017), and most importantly, decrease carbon emissions (Qin et al., 2013; Chai et al., 2014; Wang X. et al., 2021). Among various cropping systems, maize-based intercropping models, such as maize-soybean, maize-wheat, maize-rape, maize-potato, and maize-pea can achieve lower carbon emissions compared to monocropping patterns (Chai et al., 2014; Shen et al., 2018; Sun et al., 2021; Wang X. et al., 2021; Yin et al., 2022). In particular, pea/maize intercropping, widely practiced in northwestern China (Zhao et al., 2016), has been demonstrated to reduce carbon emissions by 31% compared to monoculture maize (Chai et al., 2014). Therefore, further research on mechanisms to mitigate CO_2_ emissions in the cereal-legume intercropping system has emerged as a priority point in modern agricultural production.

Intercropping improves microbial diversity and alters soil microbial community composition by indirectly changing soil properties (Song et al., 2007; Gong et al., 2019). Soil microorganisms perform various ecological functions, including N and C cycling, profoundly impacting soil productivity and sustainability (Theuerl and Buscot, 2010; Nimmo et al., 2013). With the development of bioinformatics, the diversity and structure of microorganism have been widely used to indicate their ecological function and changes in soil quality (Chu et al., 2007). Since the microorganisms involved in soil carbon degradation are extremely abundant, the composition of microbial community structures can explain soil degradation (Nannipieri et al., 2017). It is still a research hotspot to explore the role of soil microorganisms of agricultural greenhouse gas (GHG) emissions (Jiang et al., 2021). However, there is little research on how intercropping pattern reduces soil GHG emissions by altering soil microorganisms. Fertilization management, another main factor of causing the GHG on farmland, can greatly influence microbial diversity, soil properties, and enzyme activity (Luo et al., 2018; Huang et al., 2020). The application of the steel slag and biochar has altered the composition of soil bacterial communities, thus mitigating CO_2_ emissions in paddies (Chen et al., 2016; Wang et al., 2020). Moreover, biochar and controlled irrigation can be applied to mitigate GHG emissions and mediate the structures of soil microbial communities (Jiang et al., 2021). In addition, organic fertilizer combined with monotypic controlled-release urea can reduce soil CO_2_ emissions by satisfying plant and soil microbial C and N demands (Zhang et al., 2020). However, little information is available about the underlying microbial mechanisms for integrating N fertilizer into the intercropping system to reduce CO_2_ emissions in agricultural ecosystems.

The association of legume and rhizobia is an effective N_2_- fixing system that can reduce chemical fertilizer inputs and the negative impacts of agriculture on the environment. However, in practice, farmers often apply N fertilizer for pea-maize intercropping according to tactics of sole maize, which may restrict the growth of the two crops (Hu et al., 2020). Therefore, N fertilizer management needs to be optimized, which is paramount not only to meet the requirement of the early-maturing crop (pea) but also to attend to the later-maturing crop (maize). It has been demonstrated that in an optimized N management practice, the allocations at the jointing stage and 15-day post-silking stage are 1:3 and 2:2, which can boost system productivity of wheat/maize intercropping (Xu et al., 2021). According to the previous study by our team, the combination of N fertilizer management (jointing top-dress N at 45 kg N ha^−1^ and 15-day post-flowering top-dress N at 135 kg N ha^−1^) and pea/maize intercropping shows the best effect on CO_2_ emission mitigation, mainly regulating the content of inorganic N, soil moisture, and soil temperature. However, little microbial information was available on the effect of optimizing N management on the carbon emission of pea/maize intercropping.

Based on the previous studies related to soil carbon emissions and microbes in other ecosystems, we hypothesized that the delayed application of N-fertilizer could alter the carbon emission, mainly by modifying the compositions of soil bacterial communities. Thus, the objectives of this study were to (1) determine the effects of delayed N-fertilizer application and pea/maize intercropping on soil carbon emissions; (2) compare the responses of bacterial diversity and community composition to delayed N-fertilizer application and intercropping patterns; (3) evaluate the relationships between soil chemical properties, carbon emissions, and soil microbial communities.

## Materials and methods

### Site description

The experiments were conducted at the Oasis Agricultural Trial Station (37°30′N, 103°5′E, 1,776 m a.s.l.) of Gansu Agricultural University, Gansu Province, China. The long-term (1960–2020) mean annual air temperature is 7.3°C, with a mean annual sunshine duration of 2,945 h, and an accumulated temperature (above 10°C) of 2,985°C. Abundant heat and light resources provide advantages for developing intercropping, and pea/maize intercropping is the most typical intercropping pattern (Chai et al., 2021). The soil at the experimental site is Aridisol 50 and the properties of the topsoil (0–30 cm) are as follows: pH (1:2.5 soil, water) 8.0, soil organic matter 11.3 g kg^−1^, soil bulk density 1.44 g cm^−3^, total N 0.94 g kg^−1^, available phosphorous (P; Olsen-P) 29.2 mg kg^−1^, and available potassium (K; NH4OAc-extractable-K) 152.6 mg kg^−1^.

### Experimental design

The experiment was a two-factor factorial experimental design in both seasons from 2019 to 2020, including cropping pattern and delayed N fertilizer application. The cropping pattern was arranged in the main plots, including pea/maize intercropping (three rows of maize alternating with four rows of pea), sole planting of maize, and sole planting of pea. The row spacing between crops in the treatments is shown in Supplementary Figure S1. Based on the treatment, delayed application of N-fertilizer was arranged in the subplots of the experiment, including three application treatments designed according to the main growth stages of maize [i.e., pre-planting, jointing stage (V6), pre-tasseling stage (V12), and 15-day post-silking stage (R2)]. Three kinds of N-fertilizer application for sole maize were applied at the rate of 360 kg ha^−1^. The allocations at these four stages were 2:1:4:3 for N1, 2:2:4:2 for N2, and 2:3:4:1 for N3. Among them, N3 treatment was the conventional N input of maize production in the region. The application of N fertilizer for pea was at the rate of 90 kg ha^−1^, in which 80% was base applied at sowing and 20% at flowering stage [i.e., jointing stage of maize (V6)]. The application of N fertilizer for pea/maize intercropping was calculated by the bandwidth ratio. The field experiment included seven treatments with tree replicates (Supplementary Table S1).

### Field management

The maize cultivar “Xianyu 335” and the pea cultivar “Longwan 1” were used in the research. The planting densities were 90,000 and 1,800,000 plants ha^−1^ for monoculture maize and pea, respectively. For intercropping maize and pea, they were 52,000 and 760,000 plants ha^−1^, respectively. In 2019, the pea was sowed on 30 March and maize on 19 April; the pea was harvested on 9 July and maize on 27 September. In 2020, the pea was sowed on 1 April and maize on 20 April; the pea was harvested on 9 July and maize on 27 September. Maize was covered by plastic films (0.01 mm thick and 120 cm wide) that are largely adopted in arid areas to conserve water and promote maize productivity (Gan et al., 2013).

Each experimental plot for intercropping was 34.2 m^2^ (6 m × 5.7 m), and the sole cropping plot was 36 m^2^ (6 m × 6 m). Each neighboring plot has a ridge 50 cm wide and 30 cm high to eliminate potential water movement. Chemical fertilizers, such as urea (46-0-0, N-P-K) and diammonium phosphate (18-46-0, N-P_2_O_5_-K_2_O), were applied in the research. Phosphorus was applied to the soil before planting maize and pea each year, with application rates of 180 kg P_2_O_5_ ha^−1^ and 45 kg P_2_O_5_ ha^−1^, respectively. Supplemental irrigation was applied to the experimental plot through the drip irrigation method owing to low precipitation (<156 mm annually) in this region. Except for the fertilizer application, other agronomic practices were kept uniform.

### Soil sampling

At the full flowering stage of pea (PF) and silking stage of maize (MS), specifically on 5 June and 18 July 2019, five topsoil samples (0–20 cm) in an S-shaped sampling pattern were collected randomly in each field plot using an auger (5 cm in diameter) and then mixed thoroughly as a composite sample. The soil samples in the intercropping system were collected separately according to each crop strip. Then, each soil sample was sieved into two parts through a 2 mm mesh. A portion of topsoil samples was stored at −80°C for molecular analysis, while the other sample was air-dried and stored at room temperature before property analysis.

### Soil properties

Soil organic matter (SOM) was determined by oxidizing with potassium dichromate (Zhang et al., 2020). Total N (TN) was measured using an Elementar vario MACRO cube (Elementar, Hanau, Hessen, Germany; Wang et al., 2020). Soil $NO_{3}^{-}-N$ and $NH_{4}^{+}-N$ were extracted with 2 M KCl and analyzed using a continuous flow analyzer (Skalar, Breda, Netherlands; Wang J. et al., 2021). Labile organic matter (LOM) was measured by the potassium permanganate oxidation method (Xu et al., 2011). Soil catalase activity (CAT) was estimated according to the method of Nowak et al. (2004).

### Soil respiration and total carbon emissions

Soil respiration was measured using an LI-8100A system (LI-COR, 4647 Superior Street Lincoln, Nebraska United States) with a proprietary 20 cm diameter polyvinyl chloride chamber. Specifically, Rs was measured every 2 h from 8:00 a.m. to18:00 p.m. on sunny days based on 15-day intervals before pea harvest and 20-day intervals after pea harvest to monitor seasonal shifts in soil CO_2_ fluxes. The average data of measurement time represented CO_2_ fluxes for 1 day. Measurements were taken for pea and maize strips, and the average of two strips was used for Rs in the intercropping plot (Supplementary Figure S1).

The TCE was calculated based on Rs by adopting the following formula (Zhai et al., 2011):


(1) $$\mathrm{TCE}=\sum \left[\left(t_{i+1}-t_{i}\right)\frac{R_{si+1}+R_{si}}{2}\times 0.1584\times 24\right]\times 0.2727\times 10$$

where *t* is days after the sowing stage, *i* + 1 and *i* are the current and the last monitoring date, respectively; *Rs* is soil respiration (μmol CO_2_ m^−2^ s^−1^); 0.1584 converts mol CO_2_ m^−2^ s^−1^ to g CO_2_ m^−2^ h^−1^; 0.2727 converts g CO_2_ m^−2^ h^−1^ to g C m^−2^ h^−1^; and 10 and 24 converts carbon emissions from g C m^−2^ h^−1^ to kg C ha^−1^.

### Soil DNA extraction, PCR amplification, and illumina sequencing

Microbial community DNA was extracted from 0.5 g samples (fresh soil) using the FastDNA^®^ SPIN Kit for Soil (MP Biomedicals Co., Ltd., Santa Ana, CA, United States) according to the manufacturer’s instructions. The quality of DNA extraction was checked on a 1% agarose gel. The final DNA concentration and purification were determined with a NanoDrop 2000 UV–vis spectrophotometer (Thermo Scientific, Wilmington, United States).

The hypervariable region V3-V4 of the bacterial 16S rRNA gene was amplified with primer pairs 338F (5′-ACTCCTACGGGAGGCAGCAG-3′) and 806R (5′-GGACTACHVGGGTWTCTAAT-3′) by an ABI GeneAmp^®^ 9700 PCR thermocycler (ABI, CA, United States). The PCR amplification of the 16S rRNA gene was performed under the following conditions: initial denaturation at 95°C for 3 min, followed by 27 cycles of denaturing at 95°C for 30 s, annealing at 55°C for 30 s, extension at 72°C for 45 s, single extension at 72°C for 10 min, and end at 4°C. The PCR mixtures contained 4 μl of 5 × *TransStart* FastPfu buffer, 2 μl of 2.5 mM dNTPs, 0.8 μl of forward primer (5 μM), 0.8 μl of reverse primer (5 μM), 0.4 μl of *TransStart* FastPfu DNA Polymerase, 10 ng of template DNA, and 20 μl of ddH_2_O. The PCR product, extracted from 2% agarose gels, was purified by the AxyPrep DNA Gel Extraction Kit (Axygen Biosciences, Union City, CA, United States) according to the manufacturer’s instructions and quantified using Quantus™ Fluorometer (Promega, United States). The PCR reaction was performed in triplicate.

Each purified PCR was sequenced on the Illumina MiSeq PE 300 platform (San Diego, CA, United States) at Majorbio Bio-Pharm Technology Co., Ltd. (Shanghai, China). The raw reads were deposited into the NCBI Sequence Read Archive (SRA) database (Accession Number: PRJNA842905).

### Sequencing data analysis

The raw 16S rRNA sequencing reads were demultiplexed, quality-filtered using fastp version 0.20.0 (Chen et al., 2018), and merged by FLASH version 1.2.7 (Mago and Salzberg, 2011). Sequences were merged when they met the following criteria: (i) high-quality score (*Q* ≥ 50); (ii) overlapping sequences longer than 10 bp; (iii) exact barcodes and primers. Operational taxonomic units (OTUs) based on ~97% similarity were clustered using UPARSE version 7.1 (Edgar, 2013). The taxonomy of each OTU representative sequence was identified by the Ribosomal Database Project (RDP) Classifier version 2.2 against the 16S rRNA database (silva138/16s-bacteria) using a confidence threshold of 0.7 (Wang et al., 2007).

### Statistical analysis

The variance was analyzed by Duncan’s multiple range tests at *p* < 0.05 with SPSS 25.0 software (SPSS Inc., Armonk, NY, United States). The data were analyzed using a one-way analysis of variance (ANOVA) for different treatments (*p* < 0.05), including soil carbon emissions, soil properties, and microbial characteristics.

Taxonomic alpha diversity was measured by the estimated community richness (Chao 1 index) and community diversity (Shannon index) by the Mothur software package (v.1.30.2). Non-metric multi-dimensional scaling (NMDS) based on the Bray-Curtis distance was calculated using the “vegan” package (v.3.3.1) in R and selected to refer to microbial beta diversity. The relative abundance at the phylum level was performed in Circos-0.67-7. In addition, heatmap analysis based on Spearman’s correlation was applied using the “pheatmap” package in R v.3.3.1. Network analysis was completed in Cytoscape v3.7.1 (Top 50 dominant bacterial class; Spearman correlation coefficient > 0.5 and *p* < 0.05). Moreover, Origin 2021 and R language were used to draw figures.

## Results

### Seasonal dynamics of Rs

#### Pea strip

**Figure 1:**
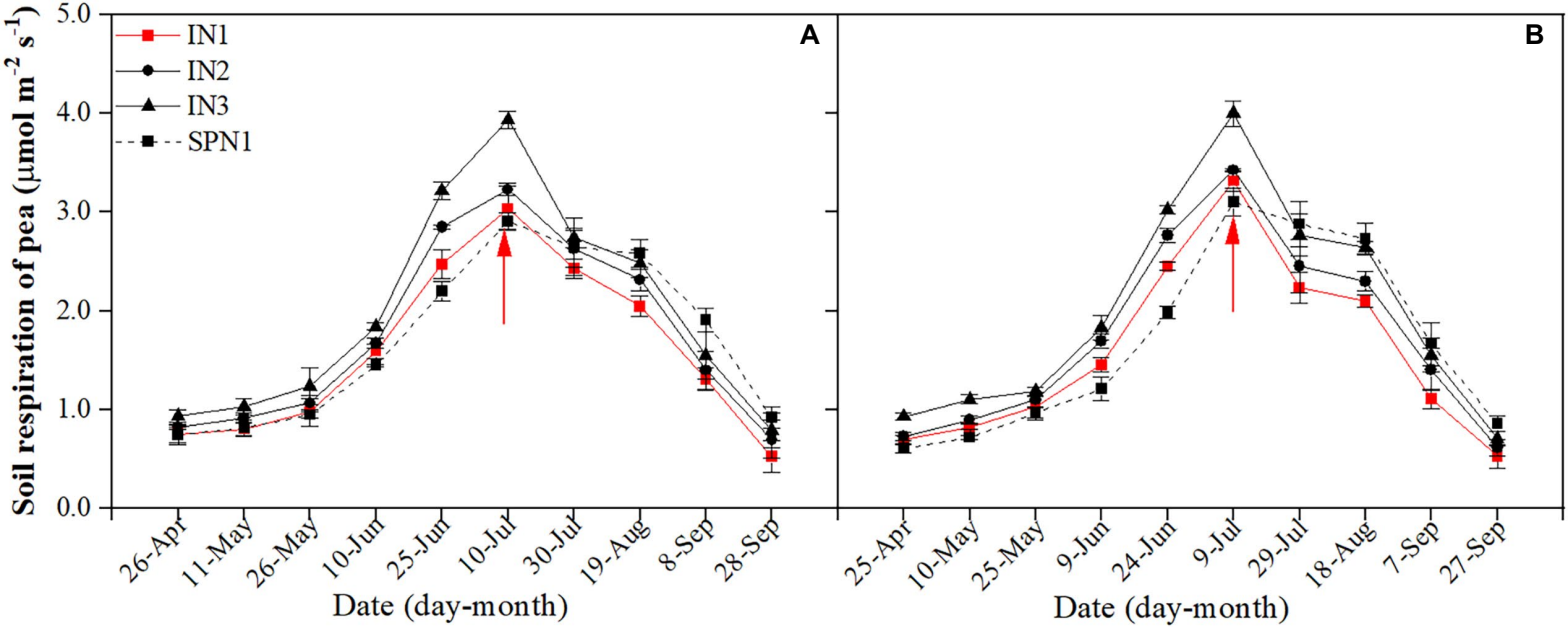
Seasonal dynamics of soil respiration of pea strips in 2019 **(A)** and 2020 **(B)**. I, pea/maize intercropping, SP, sole planting of pea. N1, N2, and N3 in the intercropping pattern represent the allocation of four-stage (sowing, jointing stage, pre-tasseling stage, and 15 days post-silking stage) was 2:1:4:3, 2:2:4:2, and 2:3:4:1, respectively. N1 in sole pattern represents 80% base fertilizer + 20% topdressing fertilizer at flowering stage. Error bars indicate the standard deviation of three replications. The red arrow shows the harvest time of pea. The description keeps uniform in the following figures.

For both research years, the seasonal variation of Rs in pea strips was typically consistent (Figure 1). The average Rs of pea strips was significantly affected by cropping patterns (*p* = 0.042), N fertilizer applications (*p* = 0.002), and the interactions of the two factors (*p* = 0.002). The influence of pea/maize intercropping on Rs differed from that of sole pea. Compared to the sole pea, the 2-year average Rs of the intercropped pea strip was increased by 22.4% during co-growth periods and was decreased by17.5% after harvesting pea. In the whole growth period, the mean Rs of intercropped pea under N1 treatment was 6.8% lower than that of sole pea, while it was 3.2% and 16.7% higher under N2 and N3, respectively.

The Rs of intercropped pea with different fertilizer N management practices did not differ among years but varied by treatments. Compared to the N3 treatment, the N1 and N2 treatments decreased the average Rs by 25.1% and 14.6% during the pea-maize co-growth period, by 23.9% and 10.2% after harvesting pea, and by 24.7% and 12.9% throughout the growth period, respectively. Compared with the intercropped pea under N1 treatment, the sole pea decreased by 9.8% in the mean Rs during the pea-maize co-growth period but increased by 24.1% after pea harvest. As a result, sole pea increased the average Rs by 6.3% throughout the growth period.

#### Maize strip

**Figure 2:**
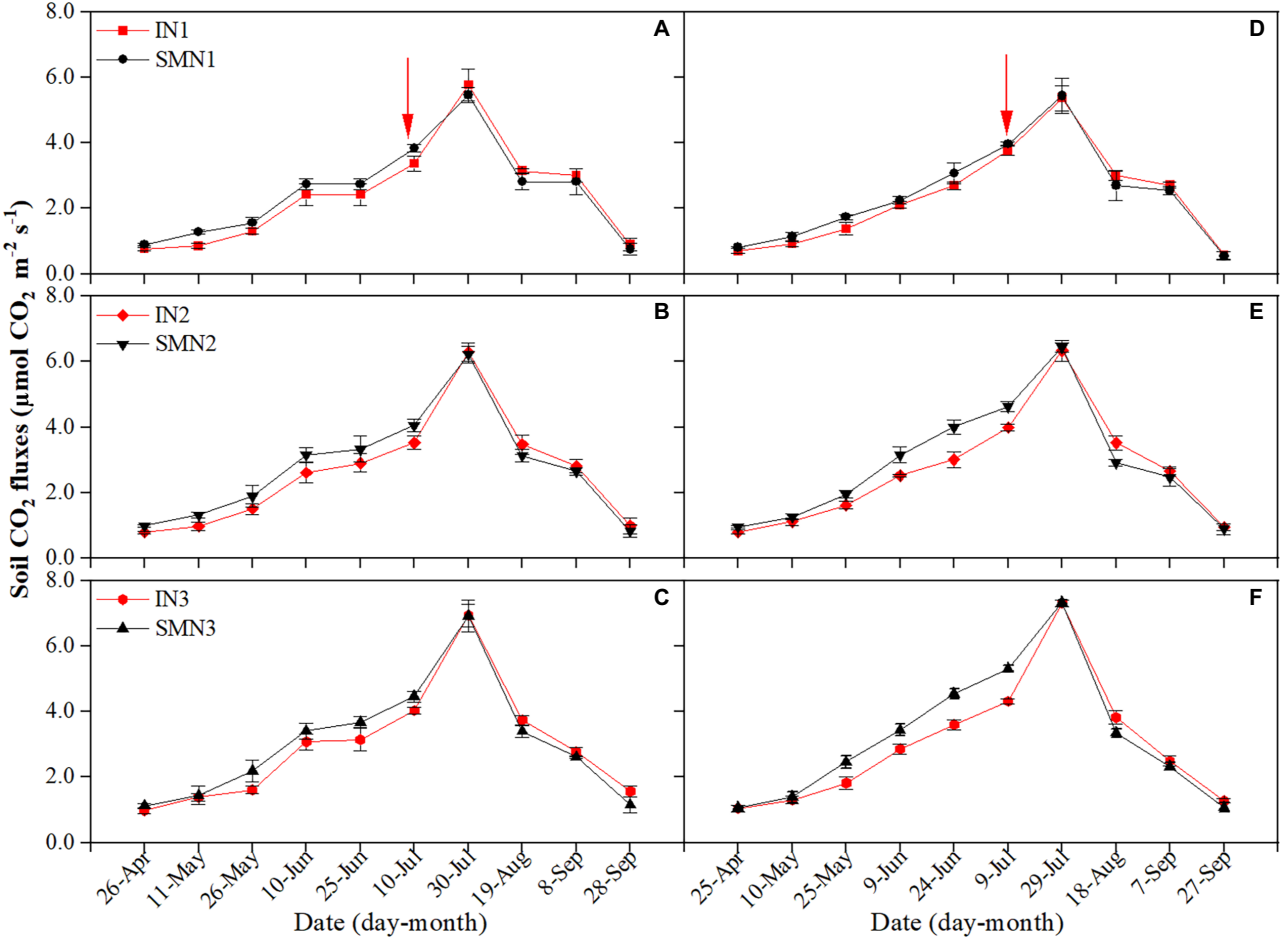
Seasonal dynamics of soil respiration of maize strips in 2019 **(A–C)** and 2020 **(D–F)**. I, pea/maize intercropping, SM, sole planting of maize. N1, N2, and N3 represent the allocation of four-stage (sowing, jointing stage, pre-tasseling stage, and 15 days post-silking stage) was 2:1:4:3, 2:2:4:2, and 2:3:4:1, respectively. Error bars indicate the standard deviation of three replications. The red arrow shows the harvest time of pea.

The average Rs of maize strips was significantly affected by cropping patterns, N fertilizer applications (*p* < 0.001), and their interaction (*p* = 0.022). The Rs of maize strips was influenced by the intercropped pea (Figure 2). During the co-growth stage, Rs of the intercropped maize was 14.6%–21.1% lower than that of monocropping while 4.1%–7.4% higher than that of monocropping after harvesting pea. Throughout the period, the Rs of intercropped maize with N1, N2, and N3 treatments was 4.0%, 7.4%, and 6.1% lower than that of monocropping maize, respectively.

A similar trend was found in both cropping patterns, with the N1 treatment featuring the lowest Rs compared to N2 and N3 treatments. During the co-growth stage, N1 and N2 treatments decreased Rs by 28.3% and 14.9%, 32.4% and 12.2% in intercropping and monocropping patterns compared to N3, respectively, with a similar reduction of 20.0% and 11.6%, 22.2% and 10.2% after pea harvest. Regarding average Rs throughout the growing season, N1 and N2 treatments reduced Rs of intercropping by 25.0% and 12.7% compared to N3, respectively, with a similar reduction of monocropping by 27.6% and 11.3%.

### Total carbon emissions

The TCE was significantly influenced by cropping systems, N management practices, and their interactions (*p* < 0.001; Table 1). By comparison the three cropping patterns, the TCE of pea/maize intercropping was 31.1%–58.8% higher than that of monoculture pea but 31.0%–35.0% lower than monoculture maize. The carbon reduction effect was highly significant in N1 and N2, compared to N3 treatment, reducing the TCE of the intercropping system by 21.1% and 10.9% and the monoculture maize by 23.2% and 9.5%, respectively.

**Table 1:** Total carbon emission (TCE; kg ha^−1^) of pea and maize in intercropping and monoculture patterns affected by N management practice in 2019 and 2020.

Treatments^a^	2019			2020		
	Maize	Pea	Total^c^	Maize	Pea	Total
Intercropping						
IN1	4,329±83e^b^	2,380 ± 48c	3,354 ± 66f	4,097 ± 61d	2,352 ± 33b	3,225 ± 27f
IN2	4,653 ± 116d	2,619 ± 19b	3,636 ± 63e	4,494 ± 91c	2,599 ± 61b	3,547 ± 71e
IN3	5,187 ± 103b	2,938 ± 112a	4,063 ± 94d	4,873 ± 47b	2,943 ± 137a	3,908 ± 87d
Monoculture						
SMN1	4,438 ± 94e	–	4,438 ± 94c	4,178 ± 142d	–	4,178 ± 142c
SMN2	4,897 ± 38c	–	4,897 ± 38b	4,802 ± 91b	–	4,802 ± 91b
SMN3	5,364 ± 76a	–	5,364 ± 76a	5,259 ± 104a	–	5,259 ± 104a
SPN1	–	2,533 ± 104bc	2,533 ± 104g	–	2,487 ± 161b	2,487 ± 161g
Significance (*p* value)						
Cropping pattern	0.001	0.020	0.000	0.000	0.036	0.000
N management practice (N)	0.000	0.001	0.000	0.000	0.007	0.000
C × N	NS^d^	0.001	0.000	0.042	0.007	0.000

a I, pea/maize intercropping, SM, sole planting of maize, SP, sole planting of pea. N1, N2, and N3 for maize represent the allocation of four-stage (sowing, jointing stage, pre-tasseling stage, and 15 days post-silking stage) was 2:1:4:3, 2:2:4:2, and 2:3:4:1, respectively. N1 for pea represents 80% base fertilizer +20% topdressing fertilizer at flowering stage. The description keeps uniform in the following tables.

b In the same column, the different lowercase letters indicate significant differences in the same year (*p* < 0.05).

c The total carbon emission of pea/maize intercropping was calculated by the average of pea strip and maize strip.

d NS indicates not significant at *p* < 0.05.

In terms of TCE in pea and maize strips of the intercropping pattern, maize strips emitted 63.5% more carbon than pea strips, demonstrating that maize strips contribute more to TCE in the intercropping pattern. Additionally, the TCE of the three cropping patterns showed that the contribution before pea harvest was lower than that after harvest (Supplementary Figure S2). In the intercropping pattern, the TCE before pea harvest accounted for 41.1% and 58.8% after pea harvest. The N1 and N2 treatments decreased the TCE of the intercropping pattern by 27.9% and 14.4% before pea harvest and by 16.7% and 8.6% after harvest, compared to N3 treatment, respectively.

### Soil properties of pea and maize strip

#### Pea strip

The soil $Ν{Ο}_{3}^{-}-Ν$, $Ν{Η}_{4}^{+}-Ν$, TN, and CAT at the PF stage were significantly influenced by the cropping system, N management practices, and their interactions, while just CAT was influenced at the MS stage (*p* < 0.05; Table 2). Soil $Ν{Ο}_{3}^{-}-Ν$, $Ν{Η}_{4}^{+}-Ν$, TN, and CAT were increased by 31.2%, 19.1%, 5.7%, and 25.0% in the intercropping pattern compared with sole pea at the PF stage but were decreased by 10.4%, 31.8%, 5.2%, and 14.1% at the MS stage. The pea/maize intercropping pattern increased soil SOM and LOM compared with monocropping plots during the period. In the pea/maize intercropping pattern, different levels of N application influenced the soil properties, which was remarkably significant at the high fertilizer level (N3) than at the low fertilizer level (N1).

**Table 2:** Soil chemical properties of pea strips under pea/maize intercropping (I) and sole cropping (S) at different N application treatments.

Period	Treatments^a^	$Ν{Ο}_{3}^{-}-Ν$ (mg/kg)	$Ν{Η}_{4}^{+}-Ν$ (mg/kg)	TN (g/kg)	SOM (g/kg)	LOM (g/kg)	CAT (0.01 mol/L KMnO_4_ ml/g)
PF	IN1	19.5de^b^	6.43bc	0.85cd	9.7ab	2.25bc	1.34c
	IN2	21.7cd	6.97b	0.893ab	10.6ab	2.50ab	1.53b
	IN3	24.3abc	9.04a	0.909a	11.6a	2.66a	1.82a
	SPN1	16.6e	6.28c	0.836cde	9.2b	1.63e	1.25d
Significance (*p* value)							
Cropping system (C)		0.000	0.000	0.000	0.002	0.000	0.000
N management practice (N)		0.022	0.000	0.000	NS	NS	0.000
C × N		0.022	0.000	0.000	NS	NS	0.000
MS	IN1	23.0bc	3.96f	0.798e	9.6b	1.51ef	0.97f
	IN2	23.7abc	4.14ef	0.824de	10.0ab	1.85de	1.05e
	IN3	26.2ab	4.65e	0.854bcd	10.8ab	2.10cd	1.18d
	SPN1	26.7a	5.58d	0.868bc	9.4b	1.20f	1.21d
Significance (*p* value)							
Cropping system (C)		0.017	0.000	0.020	NS	0.000	0.000
N management practice (N)		NS^c^	NS	NS	NS	NS	0.000
C × N		NS	NS	NS	NS	NS	0.000

a N1, N2, and N3 for intercropping pattern represent the allocation of four-stage (sowing, jointing stage, pre-tasseling stage, and 15 days post-silking stage) was 2:1:4:3, 2:2:4:2, and 2:3:4:1, respectively. N1 for sole pattern represents 80% base fertilizer +20% topdressing fertilizer at flowering stage.

b In the same column, the same lowercase letters indicate significant differences (*p* < 0.05).

c NS, not significant at the *p* < 0.05 level.

#### Maize strip

The cropping patterns significantly influenced $Ν{Ο}_{3}^{-}-Ν$, $Ν{Η}_{4}^{+}-Ν$, SOM, and CAT (*p* < 0.05), and N fertilizer application significantly influenced all soil properties at the PF stage (*p* < 0.05; Table 3). At the MS stage, cropping patterns and N fertilizer application significantly influenced LOM and CAT (*p* < 0.05). Their interaction did not influence soil properties during the period (*p* > 0.05). In general, compared to the sole maize, the intercropping pattern decreased the contents of $Ν{Ο}_{3}^{-}-Ν$ and $Ν{Η}_{4}^{+}-Ν$ by 11.1% and 15.4% at the PF stage and by 12.4% and 9.4% at the MS stage, respectively. However, the content of TN, SOM, LOM, and CAT in intercropping pattern were 1.7%, 7.5%, 10.6%, and 33.5% lower than that of sole maize at the PF stage but were 3.5%, 2.6%, 3.2%, and 13.5% higher at the MS stage. The influence of different levels of N application on soil properties was consistent, with the N1 level being lower than N2 and N3. On average, N1 treatment decreased the content of $Ν{Ο}_{3}^{-}-Ν$, $Ν{Η}_{4}^{+}-Ν$, TN, SOM, LOM, and CAT in pea/maize intercropping by 22.0%, 13.7%, 5.1%, 14.0%, 5.4%, and 27.4% compared to N3, respectively. Similarly, it decreased by 16.6%, 9.4%, 7.5%, 7.7%, 4.6%, and 26.5% in the monoculture maize, respectively.

**Table 3:** Soil chemical properties of maize strips under two cropping systems [pea/maize intercropping (I) and sole cropping (S)] and at different N application treatments.

Period	Treatments^a^	$Ν{Ο}_{3}^{-}-Ν$ (mg/kg)	$Ν{Η}_{4}^{+}-Ν$ (mg/kg)	TN (g/kg)	SOM (g/kg)	LOM (g/kg)	CAT (0.01 mol/L KMnO_4_ ml/g)
PF	IN1	17.0e^b^	5.38f	0.827cde	9.2d	1.57f	1.71 g
	IN2	19.0de	5.63ef	0.866abcd	9.4 cd	1.77ef	1.82efg
	IN3	19.8de	6.28de	0.887ab	10.5abcd	2.09 cd	2.08de
	SMN1	19.0de	6.25de	0.839bcde	9.9bcd	1.77ef	2.24 cd
	SMN2	21.0cde	6.69 cd	0.879abc	10.6abcd	1.99de	2.45bc
	SMN3	22.0bcd	7.00bc	0.907a	10.8abcd	2.25bcd	2.81a
Significance (*p* value)							
Cropping system (C)		0.018	0.000	NS	0.014	NS	0.000
N management practice (N)		0.023	0.003	0.006	0.012	0.004	0.000
C × N		NS^c^	NS	NS	NS	NS	NS
MS	IN1	19.5de	6.71 cd	0.797ef	10.8abcd	2.59a	2.00def
	IN2	22.8abcd	7.04bc	0.811e	11.5ab	2.39ab	2.36c
	IN3	24.8abc	7.47ab	0.820de	12.2a	2.30bc	2.66ab
	SMN1	22.9abcd	7.53ab	0.753f	10.8abcd	2.41ab	1.75 fg
	SMN2	25.6ab	7.59ab	0.785ef	11.1abc	2.34abc	2.06de
	SMN3	26.8a	8.09a	0.805ef	11.6ab	2.29bc	2.24 cd
Significance (*p* value)							
Cropping system (C)		NS	0.006	NS	NS	0.028	0.003
N management practice (N)		0.040	NS	NS	NS	0.001	0.001
C × N		NS	NS	NS	NS	NS	NS

a N1, N2, and N3 represent the allocation of four-stage (sowing, jointing stage, pre-tasseling stage, and 15 days post-silking stage) was 2:1:4:3, 2:2:4:2, and 2:3:4:1, respectively.

b In the same column, the same lowercase letters indicate significant differences (*p* < 0.05).

c NS, not significant at the *p* < 0.05 level.

### Bacterial community diversity

#### Bacterial alpha diversity

After quality sequencing, both bacterial communities (a total of 1,271,742,085 sequences) were obtained using the 338F/806R (bacterial 16S rRNA) primer sets in all soil samples. The number of bacterial sequences ranged from 213 to 535 per sample (mean = 416.9). The datasets were rarefied to 3,049,811 sequences for downstream analysis of bacterial sequences.

The OTU level approach was used to calculate the microbial diversity under different treatments. Variance analysis showed that cropping patterns significantly affected bacterial richness index (Chao 1) and diversity index (Shannon; *p* < 0.05; Table 4). The pea/maize intercropping pattern reduced OTUs but increased the Shannon and Chao 1 indices compared with the sole pea at the PF stage. However, the intercropping pattern increased OTUs and the Chao 1 index at the MS stage. In addition, N1 treatment in the intercropping pattern decreased OTUs, Shannon, and Chao1 indices at the PF stage compared to N3; it increased the Shannon and Chao1 indices at the MS stage.

**Table 4:** Alpha-diversity indices of the soil bacteria in pea strips under three N application treatments.

Period	Treatments	OTUs^b^	Shannon	Chao 1
PF	IPN1^a^	38,387±5,965b^c^	6.82 ± 0.05a	4,359 ± 74a
	IPN2	39,240 ± 2,648ab	6.75 ± 0.03b	4,247 ± 173ab
	IPN3	40,608 ± 1,505ab	6.84 ± 0.03a	4,384 ± 123a
	SPN1	56,180 ± 2,073ab	6.7 ± 0.01b	4,044 ± 99c
MS	IPN1	55,785 ± 2,529ab	6.74 ± 0.06b	4,174 ± 68bc
	IPN2	53,753 ± 1,608ab	6.73 ± 0.02b	4,092 ± 58bc
	IPN3	58,644 ± 8,937a	6.70 ± 0.05b	4,074 ± 43bc
	SPN1	52,662 ± 26,350ab	6.74 ± 0.06b	3,800 ± 75d
Significance (*p* value)				
Cropping system (C)		NS^d^	0.021	0.000
N management practice (N)		NS	NS	NS
C × N		NS	NS	NS

a I, pea/maize intercropping, SP, sole planting of pea. N1, N2, and N3 for intercropping pattern represent the allocation of four-stage (sowing, jointing stage, pre-tasseling stage, and 15 days post-silking stage) was 2:1:4:3, 2:2:4:2, and 2:3:4:1, respectively. N1 for sole pattern represents 80% base fertilizer +20% topdressing fertilizer at flowering stage.

b OTUs: operational taxonomic units (97% similarity).

c In the same column, the same lowercase letters indicate significant differences (*p* < 0.05).

d NS, not significant at the *p* < 0.05 level.

The cropping system significantly affected the bacteria richness index (OTUs), N management practices significantly affected the diversity index (Shannon; *p* < 0.01), and their interactions did not show an influence (*p* > 0.05; Table 5). Compared with the monocropping maize, the intercropping pattern increased the Shannon and Chao1 indices at the PF stage. However, at the MS stage, OTUs were higher under the intercropping pattern than under the monocropping pattern. Compared with the N3 treatment, N1 treatment in the intercropping pattern increased OTUs by 2.9% at the PF stage and increased OTUs and the Chao1 index by 3.7% and 0.8% at the MS stage. Additionally, OTUs and the Shannon index were 1.1% and 1.7% higher in the sole planting pattern than under N3 treatment at the PF stage, whereas the Shannon and Chao1 were 1.5% and 6.7% higher at the MS stage.

**Table 5:** Alpha-diversity indices of the soil bacteria in maize strips under three N application treatments.

Period	Treatment^a^	OTUs^b^	Shannon	Chao 1
PF	IMN1	38,925±863e^c^	6.69 ± 0.02ab	4,247 ± 97ab
	IMN2	44,883 ± 1,432cde	6.39 ± 0.10c	4,035 ± 262b
	IMN3	40,718 ± 2,772de	6.61 ± 0.07b	4,144 ± 281b
	SMN1	54,107 ± 10,396abc	6.63 ± 0.01ab	4,190 ± 97b
	SMN2	63,139 ± 4,731a	6.40 ± 0.08c	4,033 ± 81b
	SMN3	59,310 ± 5,141ab	6.62 ± 0.06ab	4,095 ± 59b
MS	IMN1	46,932 ± 5,002cde	6.69 ± 0.06ab	4,092 ± 173b
	IMN2	58,590 ± 9,296ab	6.63 ± 0.02ab	4,249 ± 142ab
	IMN3	54,759 ± 4,651abc	6.59 ± 0.08b	4,206 ± 172b
	SMN1	58,247 ± 9,782ab	6.64 ± 0.05ab	4,300 ± 78ab
	SMN2	51,202 ± 1,819bcd	6.61 ± 0.03b	4,124 ± 94b
	SMN3	50,534 ± 2,962bcd	6.73 ± 0.06a	4,494 ± 118a
Significance (*p* value)				
Cropping system (C)		0.003	NS	NS
N management practice (N)		NS^d^	0.001	NS
C × N		NS	NS	NS

a I, pea/maize intercropping, SM, sole planting of maize. N1, N2, and N3 represent the allocation of four-stage (sowing, jointing stage, pre-tasseling stage, and 15 days post-silking stage) was 2:1:4:3, 2:2:4:2, and 2:3:4:1, respectively.

b OTUs: operational taxonomic units (97% similarity).

c In the same column, the same lowercase letters indicate significant differences (*p* < 0.05).

d NS, not significant at the *p* < 0.05 level.

#### Bacterial beta diversity

**Figure 3:**
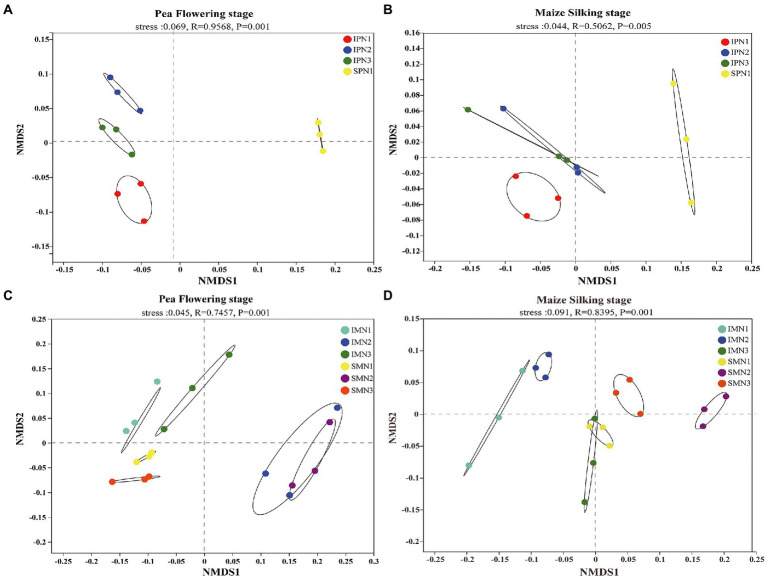
Changes in soil bacterial beta diversity (NMDS) of pea strips **(A,B)** and maize strips **(C,D)** under different treatments. I, pea/maize intercropping, SP, sole planting of pea, SM, sole planting of maize. N1, N2, and N3 for maize represent the allocation of four-stage (sowing, jointing stage, pre-tasseling stage, and 15 days post-silking stage) was 2:1:4:3, 2:2:4:2, and 2:3:4:1, respectively. N1 in sole pea represents 80% base fertilizer + 20% topdressing fertilizer at flowering stage.

The NMDS analysis was conducted to reflect microbial beta diversity (Figure 3). An evident separation of soil bacteria in the pea strip between intercropping and sole planting was observed in the NMDS plot (Figures 3A,B), suggesting the changed community composition by cropping mode. Moreover, the NMDS ordination plot showed that the bacterial community composition varied greatly among treatments at the two growth stages. For instance, N1 and N2 were separated from N3 at the PF stage, whereas just N1 was separated from N3 at the MS stage. An evident separation of soil bacteria in maize strips with different N applications was observed in the NMDS plot (Figure 3C; for instance, the separation of N1 and N3 from N2 at the PF stage), suggesting the changed community composition by N managements. However, a similar trend was not found at the MS stage (Figure 3D).

### Bacterial community structures

#### Pea strips

**Figure 4:**
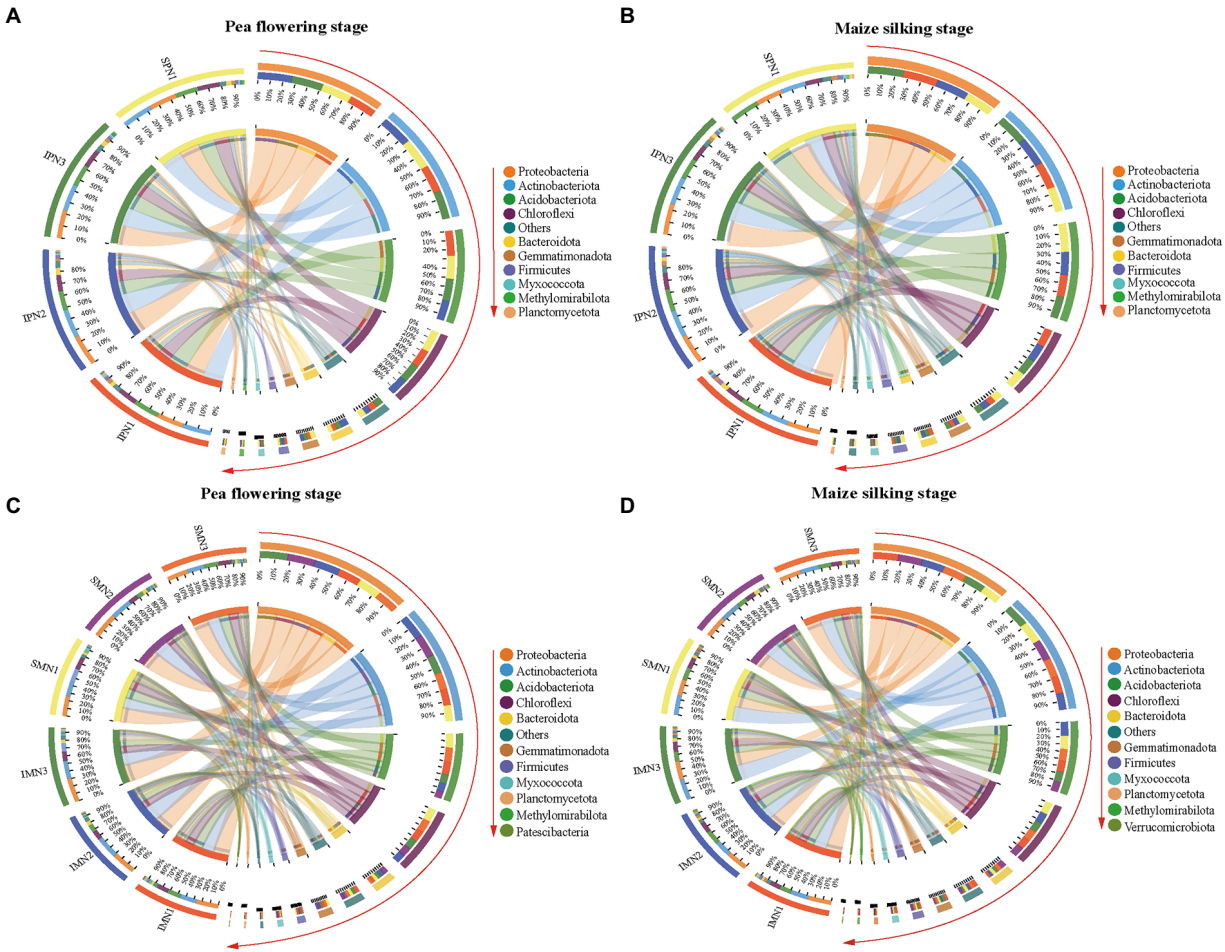
Phylum level OTUs abundance profile of bacteria 16S rRNA in pea strips **(A,B)** and maize strips **(C,D)** soils at different growth stages under different nitrogen fertilizer applications (TOP 10 in groups). I, pea/maize intercropping, SP, sole planting of pea, SM, sole planting of maize. N1, N2, and N3 for maize represent the allocation of four-stage (sowing, jointing stage, pre-tasseling stage, and 15 days post-silking stage) was 2:1:4:3, 2:2:4:2, and 2:3:4:1, respectively. N1 for sole pea represents 80% base fertilizer + 20% topdressing fertilizer at flowering stage.

The dominant bacterial phyla at the PF stage were consistent with that at the MS stage (Figure 4). Additionally, the dominant bacterial phyla in the treatments were Proteobacteria, Actinobacteriota, Acidobacteriota, Chloroflexi, Bacteroidota, Gemmatimonadota, Firmicutes, Myxococcota, Methylomirabilota, and Planctomycetota (relative abundance >1%; Figures 4A,B; Supplementary Table S2). It was noticeable that Proteobacteria, Actinobacteriota, and Bacteroidota were more abundant in the monocrop soil than in the intercropped soil at the PF stage. In contrast, Proteobacteria, Actinobacteriota, Chloroflexi, Bacteroidota, and Planctomycetota were more abundant in the intercropped soil than in the monocrop soil at the MS stage. At the class level, the relative abundance of Thermoanaerobaculia, KD4-96, Dehalococcoidia, TK10, Gitt-GS-136, and Gemmatimonadetes at the PF stage was significantly higher in the monocropping system than in the intercropping system, belonging to Acidobacteriota, Chloroflexi, and Gemmatimonadota, respectively. However, the relative abundance of Actinobacteria, Anaerolineae, and Bacteroidia at the MS stage was significantly higher in the intercropped system than in the monocropping system.

In the intercropping pattern, N1 treatment increased the relative abundance of Acidobacteriota, Acidobacteriota, Choroflexi, Gemmatimonadota, Firmicutes, and Planctomycetota at the PF stage compared with N3. Furthermore, at the class level (Supplementary Figure S3; Supplementary Table S2), the relative abundance of Acidimicrobiia, MB-A2-108, Vicinamibacteria, Blastocatellia, Anaerolineae, KD4-96, Dehalococcoidia, Gemmatimonadetes, and Bacilli were increased in N1 treatment. In addition, N1 treatment increased the relative abundance of Chloroflexia, Anaerolineae, KD4-96, Dehalococcoidia, and Bacteroidia, which belong to Choroflexi and Bacteroidota.

#### Maize strips

According to bacterial community compositions, the dominant bacterial phyla of maize were Proteobacteria, Acitinbacteriota, Acidobacteriota, Choroflexi, Bacteroidota, Gemmatimonadota, Firmicutes, and Myxococcota (relative abundance >1%; Figures 4C,D; Supplementary Table S2). The relative abundance of Proteobacteria, Gemmatimonadota, Firmicutes, and Myxococcota was increased at both sampling stages for pea/maize intercropping than for monoculture maize. Furthermore, at the class level (Supplementary Figure S3; Supplementary Table S3), the intercropping pattern increased the relative abundance of Alphaproteobacteria and Acidimicrobiia but decreased that of Vicinamibacteria, Chloroflexia, and KD4-96 compared to monoculture maize.

The comparison of different N fertilizer managements showed that the relative abundance of Proteobacteria in the intercropping pattern was decreased by the N1 treatment at the PF stage but increased at the MS stage (Supplementary Figure S3; Supplementary Table S2). However, no similar trend was found in the dominant Acidobacteriota and Myxococcota. For the class level, N1 reduced the relative abundance of Alphaproteobacteria and Gammaproteobacteria, the branch of Proteobacteria, in the intercropping system at the PF stage, and increased their abundance at the MS stage compared to N3 treatment.

### Correlation relationship among dominant class, soil properties, and Rs

**Figure 5:**
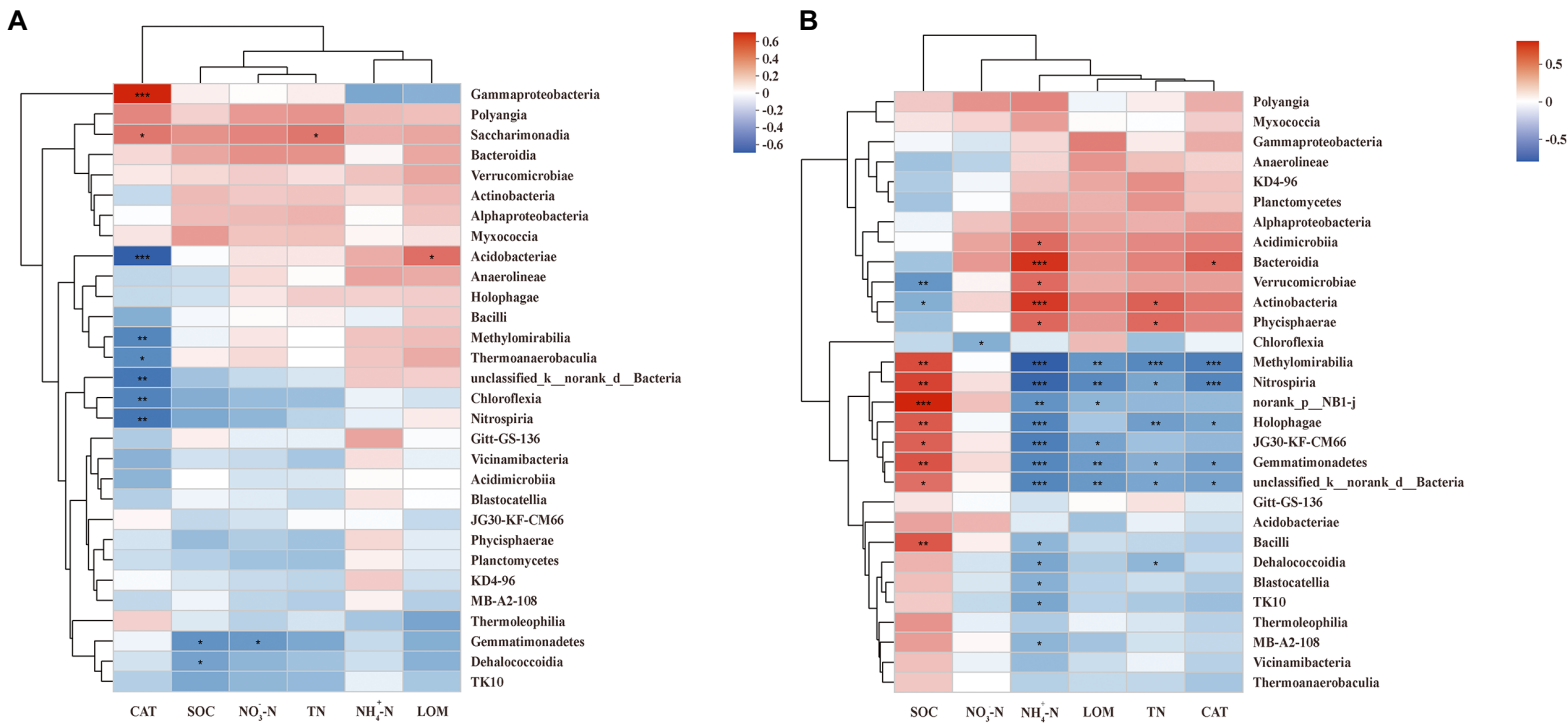
Spearman correlation heatmap of dominant class and soil properties at pea flowering stage **(A)** and maize silking stage **(B)**. SOM, soil organic matter; TN, total nitrogen; NO^-^_3_−N, nitrate; NH^+^_4_−N, ammonium; CAT, catalase activity; LOM, labile organic matter. ***, **, and * indicate *p* < 0.001, *p* < 0.01, and *p* < 0.05, respectively.

A Spearman correlation heatmap was applied to show the correlations between soil properties and dominant class. The soil properties that correlated with most soil bacteria at the PF stage were CAT (8 classes in total), followed by SOM (2), $Ν{Ο}_{3}^{-}-Ν$, TN, and LOM (1 in each; Figure 5A). Additionally, CAT showed the most negative correlation (6), mainly with Chloroflexia, Methylomirabilia, Thermoanaerobaculia, Unclassified Bacteria, Acidobacteriae, and Nitrospiria and positive correlation (2) with Gammaproteobacteria and Saccharimonadia. Moreover, $Ν{Ο}_{3}^{-}-Ν$ and SOM presented significantly negative correlations with Gemmatimonadetes. Interestingly, LOM showed a significantly positive correlation with Acidobacteriae.

The correlation between soil properties and dominant class at the MS stage differed from that at the PF stage (Figure 5B). The soil properties correlated with most soil bacteria at the MS stage were $Ν{Η}_{4}^{+}-Ν$ (17 classes in total), followed by SOM (10), TN (8), LOM and CAT (6 in each), $Ν{Ο}_{3}^{-}-Ν$ (1). In addition, $Ν{Η}_{4}^{+}-Ν$ had significantly negative correlations (12), mainly with Blastocatellia, Gemmatimonadetes, Bacilli, Methylomirabilia, Dehalococcoidia, Holophagae, Unclassified Bacteria, MB-A2-108, TK10, Nitrospiria, Uorank NB1-j, and JG30-KF-CM66. Moreover, SOM showed only significantly positive correlations except for Actinobacteria and Verrucomicrobiae; TN and CAT showed only significantly negative correlations except for Actinobacteria, Phycisphaerae, and Bacteroidia, respectively. Interestingly, LOM and $Ν{Ο}_{3}^{-}-Ν$ only presented negative correlations.

**Figure 6:**
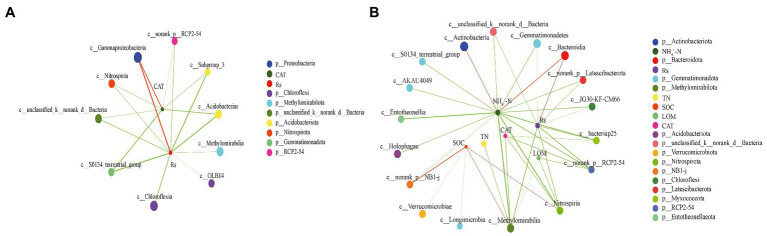
Two-factor network constructed around the main class of bacterial 16S rRNA at pea flowering stage **(A)** and maize silking stage **(B)** based on significant Spearman rank correlation coefficients. The circles of different colors indicate different classes, circle size indicates the taxonomic abundance; red and green line indicate positive and negative correlation, respectively; line thickness represents the strength of the correlation, with thicker lines indicating stronger correlations.

To identify the relationships between the main bacterial classes and environmental factors, network analyses were conducted between the top 50 microbial classes, soil properties, and Rs to explore interactions (Figure 6). The complexity of the network increased from the PF stage (number of nodes = 12, number of edges = 17, average path length = 1.787, and diameter = 3) to the MS stage (number of nodes = 23, number of edges = 37, average path length = 2.300, and diameter = 4). The network at the PF stage showed that Rs had strong positively significant correlations with Gammaproteobacteria belonging to Proteobacteria (*r* = 0.752, *p* < 0.01). Additionally, Rs showed negatively significant correlations with Acidobacteriae, Subgroup_5 (belonged to Acidobacteriota), and S0134_terrestrial_group (belonging to Gemmatimonadota; |*r*| > 0.7, *p* < 0.01). However, the Rs at the MS stage only showed positively significance with Bacteroidia (*r* = 0.562, *p* < 0.01). In contrast, Rs had a significantly negative correlation with Gemmatimonadetes, Methylomirabilia, Unclassified Bacteria, Nitrospiria, JG30-KF-CM66, Norank Latescibacterota, bacteriap25, and Norank RCP2-54 (|*r*| > 0.5, *p* < 0.01). Most importantly, the Rs at PF stage and MS stage were negatively significant correlated with the Chloroflexia (|*r*| = 0.646) and JG30-KF-CM66 (|*r*| = 0.519), which were both belong to Chloroflexi.

## Discussion

### Impacts of delayed N fertilizer application on the CO_2_ emission of pea/maize intercropping systems

Agricultural soil has been considered as the “source” of atmospheric CO_2_ since its beginning (Yang et al., 2021). Optimization agronomy measures to decrease soil Rs from farmland acts as a critical strategy in mitigating CO_2_ emissions in agricultural production (Gan et al., 2011). Previous studies demonstrated that intercropping patterns could significantly mitigate the Rs rate from the production of field crops (Chai et al., 2014; Yang et al., 2021). Moreover, Rs serves as an effective way to measure carbon emissions from soil (Raich and Tufekciogul, 2000). In the present study, the Rs of intercropping pea was significantly higher than that of monoculture during the co-growth stage, while that of intercropping maize was significantly lower than that of monoculture. This phenomenon could be explained by three driving factors. Firstly, pea, an early-maturing, cool-season crop, had a higher competitive edge than maize, a late-maturing, warm-season crop, thereby intercropped pea had a relatively higher competition for resources (Hu et al., 2016). Secondly, applying legumes to the intercropping system could reduce the application of N fertilizer for intercropping systems owing to symbiotic N_2_ fixation, thus reducing carbon emission compared to a monoculture (Hauggaard-Nielsen et al., 2016; Hu et al., 2017). Finally, intercropped maize would not be suppressed by the interspecific competition and usually exhibited compensatory growth when pea was harvested, so as to increase carbon emission (Zhao et al., 2019). Therefore, pea/maize intercropping was an effective strategy to achieve carbon emission reduction.

The application of N fertilizers has been commonly adopted to boost crop yield, and excessive application is a crucial influencing factor of GHG in farmland (Yao et al., 2012; Chen et al., 2014; Daly and Hernandez-Ramirez, 2020). The most direct practice to lower GHG is to reduce the amount of N fertilizer applied to farmland. However, when the N fertilizer was not applied to farmland, CO_2_ emissions unexpectedly significantly increased (Wang X. et al., 2021), indicating that an excessive reduction of N fertilizer was not a good strategy for mitigating CO_2_ emissions in agricultural production. On the premise of the same amount of N application, optimizing N fertilizer management was a possible measure to reduce GHG emissions in farmland. In the present study, delayed application of N fertilizer could decrease the Rs rate of the pea/maize intercropping system, which was consistent with the results in previous study that optimized management practices of N fertilizer could mitigate CO_2_ emission (Zhang et al., 2021). One possible reason was that the increasing root respiration and root C inputs as a consequence of N fertilization stimulated soil CO_2_ emission (Bicharanloo et al., 2020; Zhang et al., 2020). Another possible reason was that N fertilizer offers C and N substrates as the primary energy sources for microorganism growth, thus stimulating CO_2_ emissions (Zang et al., 2016). Furthermore, the delayed application of N fertilizer could match fertilizer N supply with crop N requirement, which were crucial to achieve high productivity and mitigate carbon emission (Chai et al., 2014; Hu et al., 2020). Further studies were needed to explore the potential mechanism of mitigating carbon emissions in pea/maize intercropping by delayed application of N fertilizer.

### Effects of delayed N fertilizer application on soil bacterial diversity and community structures

Bacteria are the most abundant and dominant of the primary soil microbes in terms of biodiversity and their influence on essential soil processes (Gong et al., 2019). In the present study, the bacterial richness and diversity of intercropped pea and intercropped maize was higher than the corresponding monoculture at the PF stage, while the same trend was not observed at the MS stage. This was probably attributed to the enhancement of crop diversity during the co-growth stage and the strong interspecific relationship, which promoted the growth of soil microorganisms, thus increasing the bacterial diversity and abundance. However, delayed N fertilizer application did not show a positive effect at this stage compared to the conventional N management. Soil nutrient supply capacity was a critical impact factor for soil microbial diversity (Fu et al., 2019). Soil properties might explain the variations. The delayed N fertilizer application decreased the amount of N fertilizer at the first topdressing compared with the conventional N management. Meanwhile, conventional N management substantially enhanced soil properties which promoted the growth of microorganisms. Previous study had similarly demonstrated that soil properties are improved in N fertilizer application, thereby promoting the growth of microorganisms (Xue et al., 2006). Both cropping patterns and N management influenced the bacterial beta diversity of pea strips, but only the latter affected maize strips. The major reason was that the intercropping pattern changed the soil microenvironment and nutrient content, thus affecting the growth of the soil microorganisms (Cuartero et al., 2022). Moreover, fertilization management regimes could affect microbial beta diversity by altering soil properties, including soil pH, bulk density, electrical conductivity, water content, total nitrogen, organic carbon, $Ν{Ο}_{3}^{-}-Ν$, and available potassium (Ren et al., 2021). Therefore, the soil bacterial diversity was regulated by the cropping patterns and N fertilizer application managements, but the regulation varied with the crops and the growth period.

Changes in bacterial community composition are usually used to reflect nutritional contents and structural features of soils (Mouhamadou et al., 2013). Our analysis revealed that the abundance of Bacteroidota and Chloroflexi in pea strips were regulated by intercropping pattern, but delayed N fertilizer application just increased the abundance of Chloroflexi. The relative abundance of Proteobacteria in intercropped maize was increased at two sampling stages, but increased by the delayed N fertilizer application at MS stage. The universality and importance of these phyla have been demonstrated in previous research (Fu et al., 2019; Guo et al., 2021). These results were consistent with other cereal-legume intercropping system (Gong et al., 2019). The main reason for this phenomenon was the combination of delayed N fertilizer application and the pea/maize intercropping pattern could alter soil properties, thus regulating the structure of bacterial communities. Changes in the chemical properties and nutrient status of soil, mainly the CAT activity at the PF stage and ammonium at the MS stage, are the most important factors causing differences in microbial community structure. Consequently, the composition of bacteria was stimulated by enzyme activities and soil properties with considerable change under cereal-legume intercropping systems (Gong et al., 2019; Yu et al., 2021).

### Impacts of applying the delayed N fertilizer application to pea/maize intercropping on CO_2_ emissions and the underlying mechanisms

Our results suggested that the delayed application of N fertilizer could decrease the CO_2_ under the pea/maize intercropping pattern. A previous study showed that the reduction in CO_2_ emissions was mainly due to the changes in bacterial community composition (Wang et al., 2020). Network analysis showed that the Rs rate mainly related to the phylum Proteobacteria and Chloroflexi at the PF stage and the phylum Bacteroidota and Chloroflexi at the MS stage, which were the most abundant bacterial phyla in soil environments (Guo et al., 2021; Cuartero et al., 2022). Furthermore, the relative abundance of Proteobacteria decreased with the reduction in the CAT activity, thereby mitigating the Rs rate at the PF stage. The result was probably because low N input conditions (N1) at co-growth period suppressed the growth of eutrophic bacteria, mainly Proteobacteria, thus reducing the CO_2_ emission (Ying et al., 2010). In contrast, the Rs rate at the MS stage decreased by promoting and inhibiting the growth of Chloroflexi and Bacteroidota with the reduction in ammonium content, indicating that the relationship between the Rs rate and the dominant bacterial group might depend on specific soil properties. Based on the data of environmental factors, soil bacterial community, and correlation network analysis, these bacterial groups could be promising biological indicators in soil carbon emissions. Moreover, the results revealed that the combined system could alter soil properties and these bacterial groups, thereby mitigating CO_2_ emissions. Further research was needed to regard the overall effect of this N management practice on the bacteria community by increasing sampling time.

## Conclusion

This study provided previously unavailable information on the correlation between bacterial communities and CO_2_ fluxes in the pea/maize intercropping system. Soil CO_2_ emissions were lower in the delayed N fertilizer application than in traditional N fertilizer application. Moreover, a close relationship existed between soil properties (catalase activity and $Ν{Η}_{4}^{+}-Ν$) and the dominant group (Proteobacteria, Chloroflexi, and Bacteroidota). Furthermore, the correlation between CO_2_ fluxes and the dominant group revealed by the network might imply the mitigation of CO_2_ emissions by suppressing the growth of Proteobacteria and Bacteroidota but promoting the growth of Chloroflexi. In conclusion, our results supported the hypothesis that soil CO_2_ emissions were influenced by delayed N fertilizer application and cropping patterns, mainly affected the bacterial communities and properties of soils. Further research of these interactions may provide a new horizon for achieving synergy between cropping patterns and N fertilization to mitigate GHG emissions.

## Data availability statement

The datasets presented in this study can be found in online repositories. The names of the repository/repositories and accession number(s) can be found at: https://www.ncbi.nlm.nih.gov/, PRJNA842905.

## Author contributions

QC designed the experiment. KX carried out the experiment, performed analysis, and wrote the manuscript. FH, ZF, and WY assisted to design the experiments. YN and QW reviewed the manuscript. All authors contributed to the article and approved the submitted version.

## Funding

This work was supported by the National Key Research and Development Program of China (2021YFD1700202-02), the ‘Double First-Class’ Key Scientific Research Project of the Education Department in Gansu Province (GSSYLXM-02), the Natural Science Foundation of China (U21A20218 and 32160765), and the Science and Technology Project of Gansu Province (21JR7RA836 and 20JR5RA037).

## Conflict of interest

The authors declare that the research was conducted in the absence of any commercial or financial relationships that could be construed as a potential conflict of interest.

## Publisher’s note

All claims expressed in this article are solely those of the authors and do not necessarily represent those of their affiliated organizations, or those of the publisher, the editors and the reviewers. Any product that may be evaluated in this article, or claim that may be made by its manufacturer, is not guaranteed or endorsed by the publisher.

## References

[ref1] Bicharanloo B. Shirvan M. B. Keitel C. Dijkstra F. A. (2020). Rhizodeposition mediates the effect of nitrogen and phosphorous availability on microbial carbon use efficiency and turnover rate. Soil Biol. Biochem. 142:107705. doi: 10.1016/j.soilbio.2020.107705

[ref2] Chai Q. Nemecek T. Liang C. Zhao C. Yu A. Z. Coulter J. A.. (2021). Integrated farming with intercropping increases food production while reducing environmental footprint. Proc. Natl. Acad. Sci. U. S. A. 118:e2106382118. doi: 10.1073/pnas.2106382118, PMID: 34518225 PMC8463858

[ref3] Chai Q. Qin A. Z. Gan Y. T. Yu A. Z. (2014). Higher yield and lower carbon emission by intercropping maize with rape, pea, and wheat in arid irrigation areas. Agron. Sustain. Dev. 34, 535–543. doi: 10.1007/s13593-013-0161-x

[ref4] Chen X. Cui Z. Fan M. Vitousek P. Zhao M. Ma W.. (2014). Producing more grain with lower environmental costs. Nature 514, 486–489. doi: 10.1038/nature13609, PMID: 25186728

[ref5] Chen J. Sun X. Li L. Liu X. Zhang B. Zheng J.. (2016). Change in active microbial community structure, abundance and carbon cycling in an acid rice paddy soil with the addition of biochar. Eur. J. Soil Sci. 67, 857–867. doi: 10.1111/ejss.12388

[ref6] Chen S. F. Zhou Y. Q. Chen Y. R. Gu J. (2018). Fastp: an ultra-fast all-in-one FASTQ preprocessor. Bioinformatics 34, i884–i890. doi: 10.1093/bioinformatics/bty560, PMID: 30423086 PMC6129281

[ref7] Chu H. Lin X. Fujii T. Morimoto S. Yagi K. Hu J.. (2007). Soil microbial biomass, dehydrogenase activity, bacterial community structure in response to long-term fertilizer management. Soil Biol. Biochem. 39, 2971–2976. doi: 10.1016/j.soilbio.2007.05.031

[ref8] Cuartero J. Pascual J. A. Vivo J. M. Özbolat O. Sánchez-Navarro V. Egea-Cortines M.. (2022). A first-year melon/cowpea intercropping system improves soil nutrients and changes the soil microbial community. Agric. Ecosyst. Environ. 328:107856. doi: 10.1016/j.agee.2022.107856

[ref9] Daly E. J. Hernandez-Ramirez G. (2020). Sources and priming of soil N_2_O and CO_2_ production: nitrogen and simulated exudate additions. Soil Biol. Biochem. 149:107942. doi: 10.1016/j.soilbio.2020.107942

[ref10] Edgar R. C. (2013). UPARSE: highly accurate OTU sequences from microbial amplicon reads. Nat. Methods 10, 996–998. doi: 10.1038/nmeth.2604, PMID: 23955772

[ref11] Fu Z. D. Zhou L. Chen P. Du Q. Pang T. Song C.. (2019). Effects of maize-soybean relay intercropping on crop nutrient uptake and soil bacterial community. J. Integr. Agric. 18, 2006–2018. doi: 10.1016/S2095-3119(18)62114-8

[ref12] Gan Y. T. Liang C. Wang X. Y. McConkey B. (2011). Lowering carbon footprint of durum wheat by diversifying cropping systems. Field Crops Res. 122, 199–206. doi: 10.1016/j.fcr.2011.03.020

[ref13] Gan Y. T. Siddique K. H. M. Turner N. C. Li X. G. Niu J. Y. Yang C.. (2013). Ridge-furrow mulching systems-an innovative technique for boosting crop productivity in semiarid rain-fed environments. Adv. Agron. 118, 429–476. doi: 10.1016/B978-0-12-405942-9.00007-4

[ref14] Gong X. W. Liu C. J. Li J. Luo Y. Yang Q. H. Zhang W. L.. (2019). Responses of rhizosphere soil properties, enzyme activities and microbial diversity to intercropping patterns on the loess plateau of China. Soil Till. Res. 195:104355. doi: 10.1016/j.still.2019.104355

[ref15] Gou F. van Ittersum M. K. Simon E. Leffelaar P. A. van der Putten P. E. L. Zhang L. Z.. (2017). Intercropping wheat and maize increases total radiation interception and wheat RUE but lowers maize RUE. Eur. J. Agron. 84, 125–139. doi: 10.1016/j.eja.2016.10.014

[ref16] Guo J. Wu Y. Q. Wu X. H. Ren Z. Wang G. B. (2021). Soil bacterial community composition and diversity response to land conversion is depth-dependent. Glob. Ecol. Conserv. 32:e01923. doi: 10.1016/j.gecco.2021.e01923

[ref17] Hauggaard-Nielsen H. Lachouani P. Knudsen M. T. Ambus P. Boelt B. Gislum R. (2016). Productivity and carbon footprint of perennial grass-forage legume intercropping strategies with high or low nitrogen fertilizer input. Sci. Total Environ. 541, 1339–1347. doi: 10.1016/j.scitotenv.2015.10.013 26479907

[ref18] Hu F. L. Gan Y. T. Chai Q. Feng F. X. Zhao C. Yu A. Z.. (2016). Boosting system productivity through the improved coordination of interspecific competition in maize/pea strip intercropping. Field Crops Res. 198, 50–60. doi: 10.1016/j.fcr.2016.08.022

[ref19] Hu F. L. Tan Y. Yu A. Z. Zhao C. Fan Z. L. Yin W.. (2020). Optimizing the split of N fertilizer application over time increases grain yield of maize-pea intercropping in arid areas. Eur. J. Agron. 119:126117. doi: 10.1016/j.eja.2020.126117

[ref20] Hu F. L. Zhao C. Feng F. X. Chai Q. Mu Y. P. Zhang Y. (2017). Improving N management through intercropping alleviates the inhibitory effect of mineral N on nodulation in pea. Plant Soil 412, 235–251. doi: 10.1007/s11104-016-3063-2

[ref21] Huang R. Wang Y. Y. Gao X. S. Liu J. Wang Z. F. Gao M. (2020). Nitrous oxide emission and the related denitrifier community: a short-term response to organic manure substituting chemical fertilizer. Ecotoxicol. Environ. Saf. 192:110291. doi: 10.1016/j.ecoenv.2020.110291, PMID: 32061984

[ref22] IPCC (2014). “Climate change 2014: Mitigation of climate change,” in *Contribution of Working Group III to the Fifth Assessment Report of the Intergovernmental Panel on Climate Change*. (Cambridge, UK and New York, NY: Cambridge University Press).

[ref23] IPCC (2018). Global Warming of 1.5°C: An IPCC Special Report on the Impacts of Global Warming of 1.5°C Above Pre-industrial Levels and Related Global Greenhouse Gas Emission Pathways, in the Context of Strengthening the Global Response to the Threat of Climate Change, Sustainable Development, and Efforts to Eradicate Poverty. World Meteorological Organization, Geneva.

[ref24] Jiang Z. W. Yang S. H. Pang Q. Q. Xu Y. Chen X. Sun X.. (2021). Biochar improved soil health and mitigated greenhouse gas emission from controlled irrigation paddy field: insights into microbial diversity. J. Clean. Prod. 318:128595. doi: 10.1016/j.jclepro.2021.128595

[ref25] Li L. Sun J. Zhang F. Li X. Yang S. Rengel Z. (2001). Wheat/maize or wheat/soybean strip intercropping: I. yield advantage and interspecific interactions on nutrients. Field Crops Res. 71, 123–137. doi: 10.1016/S0378-4290(01)00156-3

[ref26] Luo G. W. Rensing C. Chen H. Liu M. Q. Wang M. Guo S. W.. (2018). Deciphering the associations between soil microbial diversity and ecosystem multifunctionality driven by long-term fertilization management. Funct. Ecol. 32, 1103–1116. doi: 10.1111/1365-2435.13039

[ref27] Lynch J. Cain M. Frame D. Pierrehumbert R. (2021). Agriculture's contribution to climate change and role in mitigation is distinct from predominantly fossil CO_2_-emitting sectors. Front. Sustain. Food. Syst. 4:518039. doi: 10.3389/fsufs.2020.518039, PMID: 33644695 PMC7116829

[ref28] Mago T. Salzberg S. L. (2011). FLASH: fast length adjustment of short reads to improve genome assemblies. Bioinformatics 27, 2957–2963. doi: 10.1093/bioinformatics/btr507, PMID: 21903629 PMC3198573

[ref29] Mouhamadou B. Puissant J. Personeni E. Desclos-Theveniau M. Kastl E. M. Schloter M.. (2013). Effects of two grass species on the composition of soil fungal communities. Biol. Fertil. Soils 49, 1131–1139. doi: 10.1007/s00374-013-0810-x

[ref30] Nannipieri P. Ascher-Jenull J. Ceccherini M. T. Landi L. Pietramellara G. Renella G. (2017). Microbial diversity and soil functions. Eur. J. Soil Sci. 68, 12–26. doi: 10.1111/ejss.4_12398

[ref31] Nimmo J. Lynch D. H. Owen J. (2013). Quantification of nitrogen inputs from biological nitrogen fixation to whole farm nitrogen budgets of two dairy farms in Atlantic Canada. Nutr. Cycl. Agroecosystems. 96, 93–105. doi: 10.1007/s10705-013-9579-4

[ref32] Nowak J. Kaklewski K. Ligocki M. (2004). Influence of selenium on oxidoreductive enzymes activity in soil and in plants. Soil Biol. Biochem. 36, 1553–1558. doi: 10.1016/j.soilbio.2004.07.002

[ref33] Qin A. Z. Huang G. B. Chai Q. Yu A. Z. Huang P. (2013). Grain yield and soil respiratory response to intercropping systems on arid land. Field Crops Res. 144, 1–10. doi: 10.1016/j.fcr.2012.12.005

[ref34] Qu J. S. Zeng J. J. Li Y. Wang Q. Maraseni T. Zhang L. H.. (2013). Household carbon dioxide emissions from peasants and herdsmen in northwestern arid-alpine regions, China. Energy Policy 57, 133–140. doi: 10.1016/j.enpol.2012.12.065

[ref35] Raich J. W. Tufekciogul A. (2000). Vegetation and soil respiration: correlations and controls. Biogeochemistry 48, 71–90. doi: 10.1023/A:1006112000616

[ref36] Ren J. H. Liu X. L. Yang W. P. Yang X. X. Li W. G. Xia Q.. (2021). Rhizosphere soil properties, microbial community, and enzyme activities: short-term responses to partial substitution of chemical fertilizer with organic manure. J. Environ. Manag. 299:113650. doi: 10.1016/j.jenvman.2021.113650, PMID: 34481370

[ref37] Shen Y. W. Sui P. Huang J. X. Wang D. Whalen J. K. Chen Y. Q. (2018). Global warming potential from maize and maize-soybean as affected by nitrogen fertilizer and cropping practices in the North China plain. Field Crops Res. 225, 117–127. doi: 10.1016/j.fcr.2018.06.007

[ref38] Song Y. N. Zhang F. S. Marschner P. Fan F. L. Gao H. M. Bao X. G.. (2007). Effect of intercropping on crop yield and chemical and microbiological properties in rhizosphere of wheat (*Triticum aestivum L.*), maize (*Zea mays L.*), and faba bean (*Vicia faba L.*). Biol. Fertil. Soils 43, 565–574. doi: 10.1007/s00374-006-0139-9

[ref39] Sun T. Zhao C. Feng X. M. Yin W. Gou Z. W. Lal R.. (2021). Maize-based intercropping systems achieve higher productivity and profitability with lesser environmental footprint in a water-scarce region of Northwest China. Food Energy Secur. 10:e260. doi: 10.1002/fes3.260

[ref40] Theuerl S. Buscot F. (2010). Laccases: toward disentangling their diversity and functions in relation to soil organic matter cycling. Biol. Fertil. Soils 46, 215–225. doi: 10.1007/s00374-010-0440-5

[ref41] Wang X. L. Chen Y. Yang K. P. Duan F. Y. Liu P. Wang Z. G.. (2021). Effects of legume intercropping and nitrogen input on net greenhouse gas balances, intensity, carbon footprint and crop productivity in sweet maize cropland in South China. J. Clean. Prod. 314:127997. doi: 10.1016/j.jclepro.2021.127997

[ref42] Wang Q. Garrity G. M. Tiedje J. M. Cole J. R. (2007). Naive Bayesian classifier for rapid assignment of rRNA sequences into the new bacterial taxonomy. Appl. Environ. Microbiol. 73, 5261–5267. doi: 10.1128/AEM.00062-07, PMID: 17586664 PMC1950982

[ref43] Wang M. Y. Lan X. F. Xu X. P. Fang Y. Y. Singh B. P. Sardans J.. (2020). Steel slag and biochar amendments decreased CO_2_ emissions by altering soil chemical properties and bacterial community structure over two-year in a subtropical paddy field. Sci. Total Environ. 740:140403. doi: 10.1016/j.scitotenv.2020.140403, PMID: 32927559

[ref44] Wang J. B. Xie J. H. Li L. L. Luo Z. Z. Zhang R. Z. Wang L. L.. (2021). The impact of fertilizer amendments on soil autotrophic bacteria and carbon emissions in maize field on the semiarid loess plateau. Front. Microbiol. 12:664120. doi: 10.3389/fmicb.2021.664120, PMID: 34220750 PMC8249863

[ref45] Xu K. Chai Q. Hu F. L. Fan Z. L. Yin W. (2021). N-fertilizer postponing application improves dry matter translocation and increases system productivity of wheat/maize intercropping. Sci. Rep. 11:22825. doi: 10.1038/s41598-021-02345-5, PMID: 34819592 PMC8613184

[ref46] Xu M. Lou Y. Sun X. Wang W. Baniyamuddin M. Zhao K. (2011). Soil organic carbon active fractions as early indicators for total carbon change under straw incorporation. Biol. Fertil. Soils 47, 745–752. doi: 10.1007/s00374-011-0579-8

[ref47] Xue D. Yao H. Huang C. (2006). Microbial biomass, N mineralization and nitrification, enzyme activities, and microbial community diversity in tea orchard soils. Plant Soil 288, 319–331. doi: 10.1007/s11104-006-9123-2

[ref48] Yang H. W. Hu F. L. Yin W. Chai Q. Zhao C. Yu A. Z.. (2021). Integration of tillage and planting density improves crop production and carbon mitigation of maize/pea intercropping in the oasis irrigation area of northwestern China. Field Crops Res. 272:108281. doi: 10.1016/j.fcr.2021.108281

[ref49] Yao Z. S. Zheng X. H. Dong H. B. Wang R. Mei B. L. Zhu J. G. (2012). A 3-year record of N_2_O and CH_4_ emissions from a sandy loam paddy during rice seasons as affected by different nitrogen application rates. Agric. Ecosyst. Environ. 152, 1–9. doi: 10.1016/j.agee.2012.02.004

[ref50] Yin W. Chai Q. Fan Z. L. Hu F. L. Fan H. Guo Y.. (2022). Energy budgeting, carbon budgeting, and carbon footprints of straw and plastic film management for environmentally clean of wheat-maize intercropping system in northwestern China. Sci. Total Environ. 826:154220. doi: 10.1016/j.scitotenv.2022.154220, PMID: 35240178

[ref51] Yin W. Yu A. Z. Chai Q. Hu F. L. Feng F. Gan Y. T. (2015). Wheat and maize relay-planting with straw covering increases water use efficiency up to 46%. Agron. Sustain. Dev. 35, 815–825. doi: 10.1007/s13593-015-0286-1

[ref52] Ying J. Y. Zhang L. M. He J. Z. (2010). Putative ammonia-oxidizing bacteria and archaea in an acidic red soil with different land utilization patterns. Environ. Microbiol. Rep. 2, 304–312. doi: 10.1111/j.1758-2229.2009.00130.x, PMID: 23766082

[ref53] Yu L. L. Luo S. S. Gou Y. G. Xu X. Wang J. W. (2021). Structure of rhizospheric microbial community and N cycling functional gene shifts with reduced N input in sugarcane-soybean intercropping in South China. Agric. Ecosyst. Environ. 314:107413. doi: 10.1016/j.agee.2021.107413

[ref54] Zang H. D. Wang J. Y. Kuzyakov Y. (2016). N fertilization decreases soil organic matter decomposition in the rhizosphere. Appl. Soil Ecol. 108, 47–53. doi: 10.1016/j.apsoil.2016.07.021

[ref55] Zhai L. M. Liu H. B. Zhang J. Z. Huang J. Wang B. R. (2011). Long-term application of organic manure and mineral fertilizer on N_2_O and CO_2_ emissions in a red soil from cultivated maize-wheat rotation in China. J. Agric. Sci. 10, 1748–1757. doi: 10.1016/S1671-2927(11)60174-0

[ref56] Zhang K. P. Duan M. C. Xu Q. W. Wang Z. Y. Liu B. Y. Wang L. C. (2020). Soil microbial functional diversity and root growth responses to soil amendments contribute to CO_2_ emission in rainfed cropland. Catena (Amst) 195:104747. doi: 10.1016/j.catena.2020.104747

[ref57] Zhang Z. Yu Z. W. Zhang Y. L. Shi Y. (2021). Finding the fertilization optimization to balance grain yield and soil greenhouse gas emissions under water-saving irrigation. Soil Till. Res. 214:105167. doi: 10.1016/j.still.2021.105167

[ref58] Zhao C. Chai Q. Cao W. D. Whalen J. K. Zhao L. X. Cai L. J. (2019). No-tillage reduces competition and enhances compensatory growth of maize (*Zea mays L.*) intercropped with pea (*Pisum sativum L.*). Field Crops Res. 243:107611. doi: 10.1016/j.fcr.2019.107611

[ref59] Zhao C. Chai Q. Zhao Y. H. Mu Y. P. Zhang Y. Yu A. Z.. (2016). Interspecific competition and complementation is a function of N management in maize-pea intercropping systems. Crop Sci. 56, 3286–3294. doi: 10.2135/cropsci2016.03.0204

