# Supplementary material for: Delayed application of N fertilizer mitigates the carbon emissions of pea/maize intercropping via altering soil microbial diversity
Source: Front Microbiol. 2022 Sep 23;13:1002009. doi: 10.3389/fmicb.2022.1002009 (PMC9539669; doi:10.3389/fmicb.2022.1002009)
Supplement: Supplementary file 1 [file Data_Sheet_1.docx]

**Supplementary materials for**

**Delayed application of N fertilizer mitigates the carbon emissions of pea/maize intercropping via altering soil microbial diversity**

**Supplementary Tables**

**Table S1** N fertilizer allocation amount (kg ha^-1^) and postponed percentage in each treatment.

**Table S2** Relative abundances (average values and standard error) of bacterial compositions across taxonomical classification (Phyla and Class) under pea strips. Different letters indicate significant differences at *P* < 0.05 among different treatments at the same sampling stage.

**Table S3** Relative abundances (average values and standard error) of bacterial compositions across taxonomical classification (Phyla and Class) under maize strips at pea flowering stage. Different letters indicate significant differences at *P* < 0.05 among different treatments at the same sampling stage.

**Supplementary Figures**

**Fig. S1.** Agronomic processes, growth status of pea/maize intercropping systems (A), and layout of intercropping systems (B) in field experiment.

**Fig. S2.** Carbon emission for the intercropping and monoculture patterns before pea harvest and after pea harvest in 2019 and 2020. I, pea-maize intercropping, SM, sole planting of maize, SP, sole planting of pea. N1, N2, and N3 in the intercropping pattern represent the allocation of four-stage (sowing, jointing stage, pre-tasseling stage, and 15 d post-silking stage) was 2:1:4:3, 2:2:4:2, and 2:3:4:1, respectively. Different lowercase letters indicate significant differences among treatments in the same year (*P* < 0.05). Error bars indicate standard errors of means (n=3).

**Fig. S3.** Changes in bacterial taxonomic composition at the class level of pea (a and b) and maize (c and d) under three N application treatments. I, pea/maize intercropping, SP, sole planting of pea, SM, sole planting of maize. N1, N2, and N3 in the intercropping pattern represent the allocation of four-stage (sowing, jointing stage, pre-tasseling stage, and 15 d post-silking stage) was 2:1:4:3, 2:2:4:2, and 2:3:4:1, respectively. N1 in sole pea represents 80% base fertilizer + 20% at flowering stage topdressing fertilizer.

**Table S1** N fertilizer allocation amount (kg ha^-1^) and postponed percentage in each treatment.

| Treatments | Cropping pattern | Base N fertilizer^a^ | Top-dressing of N fertilizer | | | Total  N fertilizer | Postponed percentage^b^ (%) |
| --- | --- | --- | --- | --- | --- | --- | --- |
|  |  |  | Jointing | Pre-tasseling | 15 d post-silking |  |  |
| IN1 | Pea-maize | 72 | 29 | 83 | 63 | 247 | 20 |
| IN2 | Pea-maize | 72 | 50 | 83 | 42 | 247 | 10 |
| IN3 | Pea-maize | 72 | 71 | 83 | 21 | 247 | - |
| SMN1 | Sole maize | 72 | 36 | 144 | 108 | 360 | 20 |
| SMN2 | Sole maize | 72 | 72 | 144 | 72 | 360 | 10 |
| SMN3 | Sole maize | 72 | 108 | 144 | 36 | 360 | - |
| SPN1 | Sole pea | 72 | 18 | 0 | 0 | 90 | - |

^a^ The total nitrogen application amount of intercropping pattern was calculated by the proportion of each crop in the intercropping.

^b^ The postponed percentage applied only for maize.

**Table S2**

Relative abundances (average values and standard error) of bacterial compositions across taxonomical classification (Phyla and Class) under pea strips. Different letters indicate significant differences at *P* < 0.05 among different treatments at the same sampling stage.

| **Phyla** | **Class** | **PF stage**^a^ | | | | **MS stage** | | | |
| --- | --- | --- | --- | --- | --- | --- | --- | --- | --- |
|  |  | **IPN1**^b^ | **IPN2** | **IPN3** | **SPN1** | **IPN1** | **IPN2** | **IPN3** | **SPN1** |
| Proteobacteria |  | 21.25±1.12b^c^ | 25.63±2.6a | 23.7±2.54ab | 22.1±1.99b | 25.69±1.92a | 24.14±2.75a | 25.84±4.34a | 21.11±0.9a |
|  | Alphaproteobacteria | 13.04±1.18b | 14.5±0.85ab | 15.24±0.77a | 13.4±0.72b | 14.42±1.2a | 15.13±1.8a | 14.36±1.96a | 12.77±0.45a |
|  | Gammaproteobacteria | 8.21±0.08b | 9.2±1.23ab | 10.39±1.26a | 8.69±0.53ab | 11.27±0.75a | 10.71±2.54ab | 9.78±0.83ab | 8.34±0.46b |
| Actinobacteriota |  | 22.41±0.58a | 23.88±0.66a | 22.38±0.66a | 23.81±0.97a | 21.36±1.56a | 22.29±2.61a | 22.72±1.57a | 19.28±0.38a |
|  | Actinobacteria | 14.33±0.49b | 14.24±1.11b | 15.9±0.82a | 13.97±0.47b | 12.98±1.13a | 13.81±1.6a | 13.48±1.73a | 9.83±0.24b |
|  | Thermoleophilia | 1.17±0.24a | 1.24±0.34a | 0.91±0.23a | 1.15±0.34a | 4.15±0.18a | 4.26±0.16a | 4.23±0.64a | 4.75±0.31a |
|  | Acidimicrobiia | 0.96±0.06a | 0.95±0.06a | 0.87±0.07a | 0.75±0.05b | 3.22±0.31a | 3.74±0.49a | 3.43±0.57a | 3.27±0.09a |
|  | MB-A2-108 | 0.82±0.23a | 0.75±0.1a | 0.69±0.1a | 0.87±0.14a | 0.78±0.12a | 0.79±0.31b | 0.98±0.31ab | 1.34±0.2a |
| Acidobacteriota |  | 20.67±2.42a | 16.19±2.29b | 18.22±1.45ab | 18.25±0.76ab | 17.87±2.66a | 18.43±4.9a | 17.54±4.43a | 22.57±2.48a |
|  | Vicinamibacteria | 12.77±1.85a | 10.56±2.11ab | 9.54±0.78ab | 11.49±1.34b | 10.47±2.14a | 10.5±3.74a | 11.04±3.59a | 14.1±2.72a |
|  | Blastocatellia | 3.99±0.72a | 3.65±0.5a | 3.6±0.41a | 3.34±0.57a | 3.73±0.52a | 3.16±0.85a | 3.55±1.06a | 4.13±0.35a |
|  | Thermoanaerobaculia | 3.59±0.45b | 3.53±0.11b | 3.59±0.18b | 4.84±0.2a | 0.8±0.16b | 1±0.09a | 1.04±0.11a | 1±0.04a |
|  | Acidobacteriae | 3.5±0.41a | 3.71±0.34a | 3.54±0.09a | 4±0.46a | 0.72±0.03a | 0.89±0.17a | 0.8±0.1a | 0.82±0.02a |
|  | Holophagae | 0.92±0.05a | 0.94±0.09a | 0.65±0.12b | 0.76±0.11ab | 0.96±0.1a | 1.16±0.19a | 1.1±0.17a | 1.1±0.17a |
| Chloroflexi |  | 14.8±0.2b | 13.57±0.5b | 14.62±1.46b | 16.85±1.25a | 14.31±0.51a | 13.42±1.47a | 13.21±1.66a | 13.05±0.28a |
|  | Chloroflexia | 5.28±0.17ab | 5.29±0.41ab | 5±0.38b | 5.74±0.05a | 4.83±0.1a | 4.47±0.2a | 4.6±0.32a | 4.46±0.37a |
|  | Anaerolineae | 3.97±0.12a | 3.76±0.35a | 3.01±0.1b | 3.2±0.2b | 3.1±0.02a | 2.9±0.41a | 2.77±0.47a | 1.86±0.11b |
|  | KD4-96 | 2.21±0.21b | 2.34±0.25b | 2.33±0.57b | 3.34±0.36a | 2.48±0.42a | 2.27±0.73a | 2.33±0.48a | 2.37±0.41a |
|  | Dehalococcoidia | 0.92±0.06b | 0.88±0.14b | 0.84±0.06b | 1.33±0.05a | 1.15±0.06a | 1.07±0.12a | 1.09±0.14a | 1.46±0.4a |
|  | TK10 | 0.78±0.1b | 0.76±0.07b | 0.67±0.06b | 0.96±0.06a |  |  |  |  |
|  | Gitt-GS-136 | 0.62±0.06b | 0.55±0.09b | 0.75±0.15b | 1.11±0.24a | 0.72±0.03a | 0.7±0.25a | 0.73±0.17a | 0.86±0.22a |
| Bacteroidota |  | 3.75±0.18c | 6.08±1.46a | 4.7±1.64b | 3.44±0.96c | 3.77±0.47a | 3.63±0.65a | 3.72±0.76a | 2.28±0.1b |
|  | Bacteroidia | 3.69±0.19c | 4.63±0.04b | 5.98±0.96a | 3.34±0.13c | 3.71±0.45a | 3.67±0.77a | 3.6±0.66a | 2.25±0.09b |
| Gemmatimonadota |  | 3.51±0.28b | 2.9±0.45c | 3.18±0.5bc | 4.21±0.13a | 4.01±0.11b | 4.03±0.11b | 3.91±0.46b | 5.48±0.51a |
|  | Gemmatimonadetes | 2.43±0.12b | 2.24±0.13bc | 2.03±0.11c | 3.14±0.18a | 2.83±0.14b | 2.99±0.53b | 3±0.21b | 3.96±0.39a |
| Firmicutes |  | 2.82±0.28a | 2.53±0.42ab | 2.24±0.32ab | 1.97±0.53b | 2.43±0.12b | 3.12±0.75ab | 2.72±0.21b | 3.81±0.65a |
|  | Bacilli | 2.35±0.29a | 2.03±0.09a | 2.24±0.52a | 1.75±0.36a | 2.26±0.12b | 2.47±0.15b | 2.79±0.71ab | 3.47±0.63a |
| Myxococcota |  | 1.81±0.33a | 1.6±0.3a | 1.9±0.3a | 1.59±0.14a | 1.83±0.19b | 1.91±0.25b | 1.68±0.17b | 2.59±0.15a |
|  | Polyangia | 1.15±0.3a | 1.2±0.21a | 1±0.09a | 1.02±0.05a | 1.1±0.12b | 1±0.14b | 1.2±0.2b | 1.65±0.1a |
| Methylomirabilota |  | 1.4±0.13ab | 1.2±0.18b | 1.6±0.18a | 1.44±0.19ab | 1.67±0.29a | 1.88±0.01a | 1.65±0.12a | 2.25±0.53a |
|  | Methylomirabilia | 1.4±0.13ab | 1.6±0.06a | 1.2±0.19b | 1.44±0.16ab | 1.67±0.29a | 1.65±0.12a | 1.88±0.01a | 2.25±0.53a |
| Planctomycetota |  | 1.53±0.31a | 1.17±0.33a | 1.28±0.06a | 1.41±0.18a | 1.22±0.05a | 1.35±0.28a | 1.3±0.35a | 1.18±0.25a |

^a^ PF stage and MS stage represent pea flowering stage (3 June) and maize silking stage (18 July), respectively.

^b^ I, pea-maize intercropping, SP, sole planting of pea. N1, N2, and N3 in the intercropping pattern represent the allocation of four-stage (sowing, jointing stage, pre-tasseling stage, and 15 d post-silking stage) was 2:1:4:3, 2:2:4:2, and 2:3:4:1, respectively. N1 in sole pea represents 80% base fertilizer + 20% at flowering stage topdressing fertilizer.

^c^ The same lowercase letters among different treatments at the same period indicated significant differences at *P* < 0.05 using Duncan’s test.

**Table S3**

Relative abundances (average values and standard error) of bacterial compositions across taxonomical classification (Phyla and Class) under maize strips at pea flowering stage. Different letters indicate significant differences at *P* < 0.05 among different treatments at the same sampling stage.

| **Phyla** | **Class** | **PF stage**^a^ | | | | | | **MS stage** | | | | | |
| --- | --- | --- | --- | --- | --- | --- | --- | --- | --- | --- | --- | --- | --- |
|  |  | **IMN1**^b^ | **IMN2** | **IMN3** | **SMN1** | **SMN2** | **SMN3** | **IMN1** | **IMN2** | **IMN3** | **SMN1** | **SMN2** | **SMN3** |
| Proteobacteria |  | 27.69±2.68ab^c^ | 29.88±4.39ab | 32.29±4.16a | 26.89±1.5ab | 32.27±3.12a | 26.25±0.75b | 29.08±4.38a | 26.9±1.19a | 25.63±2.44ab | 21.98±1.13b | 27.99±1.23a | 26.64±1.54a |
|  | Alphaproteobacteria | 14.37±1.69ab | 16.14±3.39ab | 17.56±3.94a | 12.38±0.96b | 17.08±2.17ab | 12.79±1.19ab | 16.37±3.15a | 14.19±0.66a | 16.05±1.94a | 13.43±0.83a | 16.55±0.7a | 14.96±0.81a |
|  | Gammaproteobacteria | 13.32±0.99a | 13.74±1.14a | 14.73±0.58a | 14.51±0.71a | 15.19±1.55a | 13.46±1.27a | 12.71±1.29a | 12.71±0.56a | 9.58±0.83b | 8.55±0.35b | 11.44±0.7a | 11.69±1.03a |
| Actinobacteriota |  | 20.68±1.91bc | 29.34±1.77a | 23.85±2.44b | 19.93±0.57c | 27.86±1.36a | 21.55±2.21bc | 22.25±1.51c | 21.21±0.72c | 27.75±2.86a | 26.54±1.87ab | 26.01±1.16ab | 23.5±1.48bc |
|  | Actinobacteria | 11.66±0.97c | 21.4±2.18a | 14.4±1.33b | 11.94±0.21c | 20.19±1.28a | 12±1.08c | 12.45±0.67c | 12.43±0.71c | 18.42±1.86a | 17.9±1.56a | 18.19±1.19a | 15.42±1.01b |
|  | Thermoleophilia | 3.98±0.77a | 3.9±0.31a | 4.52±0.72a | 3.99±0.24a | 3.74±0.46a | 4.95±0.93a | 4.96±0.61a | 4.17±0.13ab | 4.51±0.96ab | 4.27±0.4ab | 3.53±0.11b | 3.78±0.34b |
|  | Acidimicrobiia | 3.96±0.4ab | 3.46±0.32ab | 4.22±0.93a | 3.12±0.22b | 3.35±0.33ab | 3.51±0.43ab | 4.04±0.74a | 3.45±0.16a | 4.07±0.44a | 3.52±0.24a | 3.66±0.18a | 3.48±0.25a |
|  | MB-A2-108 | 0.95±0.22a | 0.5±0.23b | 0.55±0.28ab | 0.72±0.13ab | 0.51±0.21b | 0.96±0.2a | 0.62±0.24ab | 0.97±0.18a | 0.64±0.29ab | 0.75±0.16ab | 0.53±0.02b | 0.7±0.04ab |
| Acidobacteriota |  | 17.04±4.09bc | 10.57±3.37cd | 11.85±5.38bcd | 18.16±2.26a | 9.11±2.86d | 17.93±2.65b | 14.42±5.4a | 18.13±2.62a | 13.3±3.94a | 17.46±1.52a | 13.12±0.62a | 15.54±2.48a |
|  | Vicinamibacteria | 10.02±3.03ab | 5.92±2.56bc | 6.67±3.9abc | 11.17±1.6a | 4.99±1.99c | 11.2±1.94a | 7.99±4.05a | 11.25±2.12a | 7.45±3.07a | 10.76±1.3a | 7.98±0.69a | 9.24±2.02a |
|  | Blastocatellia | 3.76±1.14a | 2.49±0.91a | 2.41±1.35a | 3.85±0.74a | 2.12±0.77a | 3.77±0.85a | 2.74±1.37a | 3.7±0.6a | 2.51±0.91a | 3.37±0.55a | 2.79±0.25a | 2.96±0.5a |
|  | Thermoanaerobaculia | 0.98±0.15a | 0.8±0.25ab | 0.92±0.12ab | 0.85±0.06ab | 0.62±0.17b | 0.93±0.13ab | 1.12±0.21a | 0.89±0.07a | 0.94±0.3a | 1±0.2a | 0.75±0.18a | 1.04±0.27a |
|  | Holophagae | 0.77±0.07ab | 0.5±0.05c | 0.69±0.14bc | 0.93±0.09a | 0.54±0.06c | 0.79±0.16ab | 1.06±0.04a | 0.84±0.14b | 0.95±0.07ab | 0.86±0.19ab | 0.47±0.07c | 0.81±0.07b |
| Chloroflexi |  | 14±1.66ab | 11.22±1.98c | 11.92±1.34bc | 15.99±0.71a | 11.3±1.5c | 15.69±0.46a | 12.86±1.58a | 14.13±0.71a | 13.14±1.73a | 14.59±1.34a | 12.62±0.77a | 12.93±0.56a |
|  | Chloroflexia | 5.09±0.44ab | 4.65±0.34bc | 5.05±0.06ab | 5.43±0.27a | 4.34±0.42c | 4.94±0.16ab | 4.8±0.23a | 4.5±0.43a | 4.43±0.3a | 4.81±0.34a | 4.51±0.07a | 4.58±0.25a |
|  | Anaerolineae | 3.09±0.68abc | 2.29±0.32d | 2.34±0.43cd | 3.78±0.32a | 2.64±0.37bcd | 3.36±0.19ab | 2.69±0.23b | 3.14±0.21ab | 2.94±0.61ab | 3.32±0.24a | 2.68±0.12b | 2.76±0.24ab |
|  | KD4-96 | 2.31±0.44ab | 1.78±0.85b | 1.72±0.57b | 2.71±0.12ab | 1.91±0.63b | 3.26±0.31a | 1.98±0.81a | 2.67±0.36a | 2.51±0.67a | 3.01±0.65a | 2.65±0.39a | 2.29±0.25a |
|  | Dehalococcoidia | 1.05±0.05a | 0.75±0.15b | 0.77±0.05b | 1.24±0.15a | 0.69±0.08b | 1.23±0.23a | 1.07±0.06a | 1.13±0.13a | 1.12±0.16a | 1.06±0.09a | 0.8±0.06b | 1±0.05a |
| Bacteroidota |  | 3.96±0.65bc | 6.05±1.48a | 4.94±0.43ab | 4.15±0.51bc | 6.11±0.03a | 3.55±0.24c | 3.59±0.5c | 3.65±0.22c | 4.76±0.49b | 3.98±0.22c | 7.44±0.51a | 5.21±0.4b |
|  | Bacteroidia | 3.9±0.62bc | 5.98±1.48a | 4.86±0.41ab | 4.09±0.49bc | 6.03±0.04a | 3.46±0.23c | 3.55±0.5c | 3.6±0.21c | 4.7±0.49b | 3.92±0.23c | 7.38±0.5a | 5.14±0.38b |
| Gemmatimonadota |  | 3.39±0.16abc | 2.93±0.31c | 3.18±0.11bc | 3.76±0.21a | 2.94±0.33c | 3.51±0.28ab | 4.12±0.29a | 3.39±0.15bc | 3.71±0.2b | 3.13±0.27c | 2.72±0.17d | 3.05±0.02cd |
|  | Gemmatimonadetes | 2.52±0.24ab | 2.08±0.26b | 2.4±0.22ab | 2.79±0.2a | 2.2±0.3b | 2.7±0.15a | 3.08±0.37a | 2.43±0.2b | 2.87±0.28a | 2.4±0.24b | 2.07±0.05b | 2.23±0.06b |
| Firmicutes |  | 2.92±0.78a | 1.74±0.16a | 2.77±0.91a | 1.65±0.15a | 2.18±1.25a | 1.92±0.49a | 3.52±1.17a | 2.55±0.14ab | 2.84±0.85ab | 2.63±0.42ab | 1.89±0.13b | 2.61±0.48ab |
|  | Bacilli | 2.65±0.75a | 1.59±0.18b | 2.45±0.76ab | 1.51±0.14b | 1.63±0.58ab | 1.72±0.45ab | 3.2±0.98a | 2.29±0.14ab | 2.53±0.85ab | 2.28±0.38ab | 1.66±0.13b | 2.29±0.44ab |
|  | Clostridia | 0.14±0.03a | 0.08±0.03a | 0.42±0.07b | 0.06±0.02a | 0.49±0.7a | 0.07±0.02a |  |  |  |  |  |  |
| Myxococcota |  | 1.74±0.21ab | 1.59±0.17b | 2.02±0.19a | 1.77±0.02ab | 1.73±0.09ab | 1.75±0.11ab | 2.18±0.24a | 1.75±0.15bc | 1.85±0.08abc | 1.78±0.2bc | 1.66±0.13c | 2.07±0.22ab |
|  | Polyangia | 1.06±0.17a | 0.99±0.12a | 1.18±0.13a | 1.16±0.06a | 1.16±0.12a | 1.15±0.11a | 1.31±0.14ab | 1.11±0.04c | 1.24±0.04abc | 1.19±0.09abc | 1.16±0.1bc | 1.38±0.15a |
| Planctomycetota |  | 1.37±0.32abc | 1.01±0.38bc | 0.84±0.22c | 1.51±0.34ab | 0.9±0.38bc | 1.68±0.26a | 0.94±0.18c | 1.45±0.19abc | 1.48±0.55ab | 1.8±0.1a | 1.16±0.14bc | 1.49±0.26ab |
| Methylomirabilota |  | 1.47±0.01a | 0.83±0.15c | 1.15±0.24b | 1.07±0.08b | 0.75±0.11c | 1.27±0.1ab | 1.62±0.23a | 1.35±0.24ab | 0.97±0.25c | 1.09±0.1bc | 0.58±0.09d | 1.13±0.12bc |
|  | Methylomirabilia | 1.47±0.01a | 0.83±0.15c | 1.15±0.24b | 1.07±0.08b | 0.75±0.11c | 1.27±0.1ab | 1.62±0.23a | 1.35±0.24ab | 0.97±0.25c | 1.09±0.1bc | 0.58±0.09d | 1.13±0.12bc |
| Patescibacteria |  | 0.44±0.13b | 1.06±0.15a | 0.65±0.06b | 0.68±0.11b | 1.25±0.21a | 0.69±0.09b |  |  |  |  |  |  |
|  | Saccharimonadia | 0.21±0.05c | 0.79±0.11a | 0.42±0.2a | 0.39±0.06bc | 0.9±0.17a | 0.43±0.07b |  |  |  |  |  |  |
| Verrucomicrobiota |  | 0.76±0.31a | 0.92±0.34a | 0.52±0.27a | 0.73±0.09a | 0.87±0.53a | 0.77±0.11a | 0.45±0.23b | 0.81±0.07ab | 0.69±0.39ab | 1.09±0.29a | 1.12±0.32a | 0.99±0.2a |
|  | Verrucomicrobiae | 0.52±0.17a | 0.82±0.29a | 0.55±0.28ab | 0.58±0.06a | 0.76±0.47a | 0.62±0.1a | 0.31±0.16b | 0.6±0.1ab | 0.54±0.31ab | 0.9±0.26a | 0.93±0.27a | 0.79±0.17a |

^a^ PF stage and MS stage represent pea flowering stage (3 June) and maize silking stage (18 July), respectively.

^b^ I, pea-maize intercropping, SM, sole planting of maize. N1, N2, and N3 in the intercropping pattern represent the allocation of four-stage (sowing, jointing stage, pre-tasseling stage, and 15 d post-silking stage) was 2:1:4:3, 2:2:4:2, and 2:3:4:1, respectively.

^c^ The same lowercase letters among different treatments at the same period indicated significant differences at *P* < 0.05 using Duncan’s test.


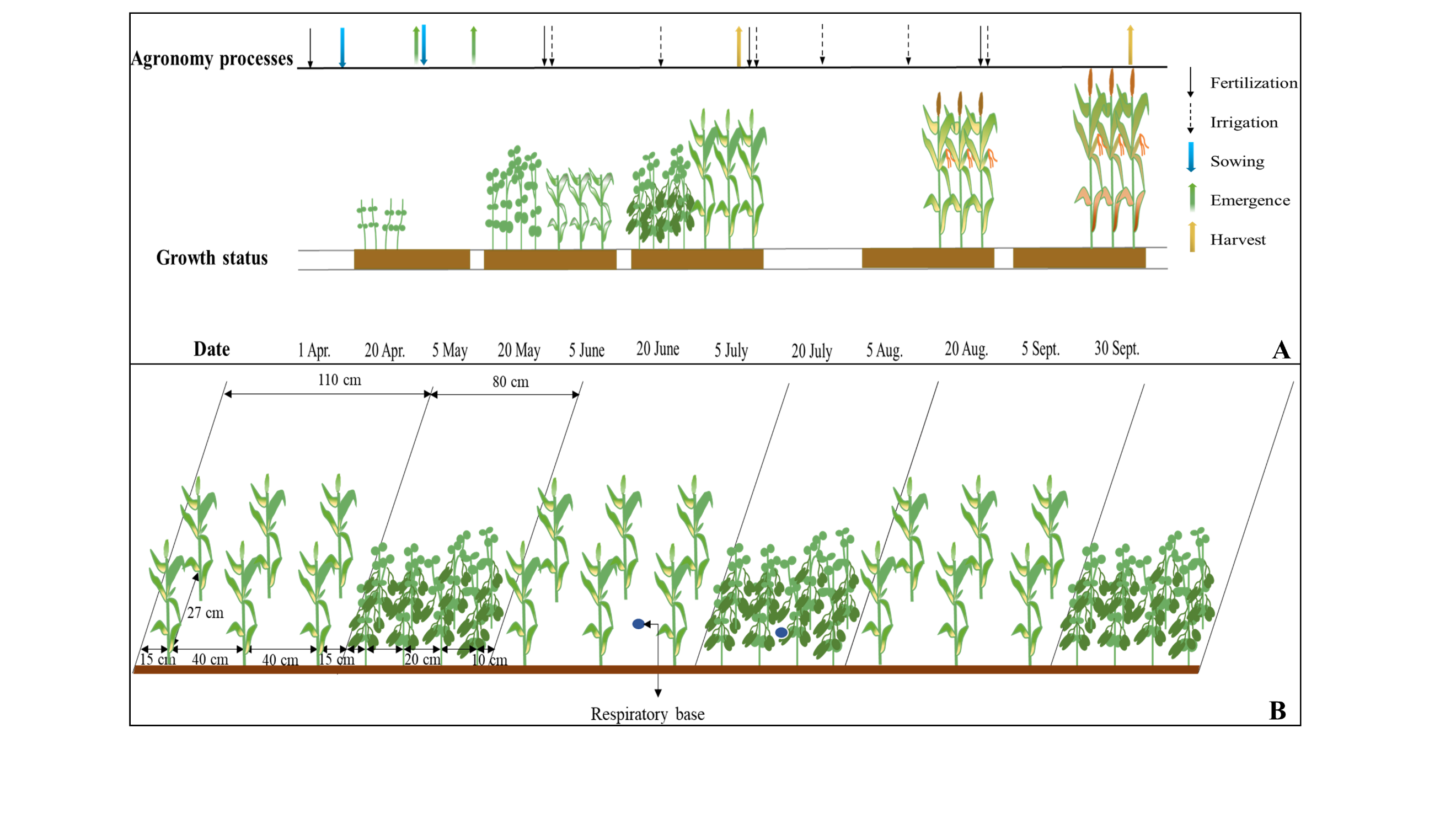


**Fig. S1.** Agronomic processes, growth status of pea/maize intercropping systems (A), and layout of intercropping systems (B) in field experiment.

**Fig. S2.** Carbon emission for the intercropping and monoculture patterns before pea harvest and after pea harvest in 2019 and 2020. I, pea-maize intercropping, SM, sole planting of maize, SP, sole planting of pea. N1, N2, and N3 in the intercropping pattern represent the allocation of four-stage (sowing, jointing stage, pre-tasseling stage, and 15 d post-silking stage) was 2:1:4:3, 2:2:4:2, and 2:3:4:1, respectively. Different lowercase letters indicate significant differences among treatments in the same year (*P* < 0.05). Error bars indicate standard errors of means (n=3).

**Fig. S3.** Changes in bacterial taxonomic composition at the class level of pea (a and b) and maize (c and d) under three N application treatments. I, pea/maize intercropping, SP, sole planting of pea, SM, sole planting of maize. N1, N2, and N3 in the intercropping pattern represent the allocation of four-stage (sowing, jointing stage, pre-tasseling stage, and 15 d post-silking stage) was 2:1:4:3, 2:2:4:2, and 2:3:4:1, respectively. N1 in sole pea represents 80% base fertilizer + 20% at flowering stage topdressing fertilizer.
